# Heparan Sulfate Sulfation by Hs2st Restricts Astroglial Precursor Somal Translocation in Developing Mouse Forebrain by a Non-Cell-Autonomous Mechanism

**DOI:** 10.1523/JNEUROSCI.1747-17.2018

**Published:** 2019-02-20

**Authors:** James M. Clegg, Hannah M. Parkin, John O. Mason, Thomas Pratt

**Affiliations:** ^1^Simons Initiative for the Developing Brain, and; ^2^Centre for Discovery Brain Sciences, Edinburgh Medical School: Biomedical Sciences, The University of Edinburgh, Edinburgh EH8 9XD, United Kingdom

**Keywords:** corpus callosum, FGF, heparan sulfate, mosaic, paracrine, telencephalon

## Abstract

Heparan sulfate (HS) is a cell surface and extracellular matrix carbohydrate extensively modified by differential sulfation. HS interacts physically with canonical fibroblast growth factor (FGF) proteins that signal through the extracellular signal regulated kinase (ERK)/mitogen activated protein kinase (MAPK) pathway. At the embryonic mouse telencephalic midline, FGF/ERK signaling drives astroglial precursor somal translocation from the ventricular zone of the corticoseptal boundary (CSB) to the induseum griseum (IG), producing a focus of *Slit2*-expressing astroglial guidepost cells essential for interhemispheric corpus callosum (CC) axon navigation. Here, we investigated the cell and molecular function of a specific form of HS sulfation, 2-O HS sulfation catalyzed by the enzyme Hs2st, in midline astroglial development and in regulating FGF protein levels and interaction with HS. *Hs2st*^−/−^ embryos of either sex exhibit a grossly enlarged IG due to precocious astroglial translocation and conditional *Hs2st* mutagenesis and *ex vivo* culture experiments show that *Hs2st* is not required cell autonomously by CC axons or by the IG astroglial cell lineage, but rather acts non-cell autonomously to suppress the transmission of translocation signals to astroglial precursors. Rescue of the *Hs2st*^−/−^ astroglial translocation phenotype by pharmacologically inhibiting FGF signaling shows that the normal role of Hs2st is to suppress FGF-mediated astroglial translocation. We demonstrate a selective action of Hs2st on FGF protein by showing that *Hs2st* (but not *Hs6st1*) normally suppresses the levels of Fgf17 protein in the CSB region *in vivo* and use a biochemical assay to show that *Hs2st* (but not *Hs6st1*) facilitates a physical interaction between the Fgf17 protein and HS.

**SIGNIFICANCE STATEMENT** We report a novel non-cell-autonomous mechanism regulating cell signaling in developing brain. Using the developing mouse telencephalic midline as an exemplar, we show that the specific sulfation modification of the cell surface and extracellular carbohydrate heparan sulfate (HS) performed by Hs2st suppresses the supply of translocation signals to astroglial precursors by a non-cell-autonomous mechanism. We further show that Hs2st modification selectively facilitates a physical interaction between Fgf17 and HS and suppresses Fgf17 protein levels *in vivo*, strongly suggesting that Hs2st acts selectively on Fgf17 signaling. HS interacts with many signaling proteins potentially encoding numerous selective interactions important in development and disease, so this class of mechanism may apply more broadly to other biological systems.

## Introduction

The corpus callosum (CC) axon tract connects the cerebral hemispheres through the corticoseptal boundary (CSB) in mice and humans and CC malformation is associated with cognitive and neurological conditions in humans (Donahoo and Richards, 2009). Precisely controlled radial glial cell (RGC) somal translocation from the ventricular zone (VZ) of the CSB to its pial surface generates midline zipper (MZ) and indusium griseum (IG) astroglial populations required for cerebral hemisphere fusion and subsequent CC axon navigation (Shu and Richards, 2001; Inatani et al., 2003; Shu et al., 2003; Smith et al., 2006; Moldrich et al., 2010; Clegg et al., 2014; Gobius et al., 2016). The movement of RGC astroglial precursors from the glial wedge (GW) to the IG (GW→IG translocation) forms an astroglial guidepost population that secretes Slit2 to guide CC axons across the telencephalic midline.

Fibroblast growth factors (FGFs) are an evolutionarily ancient family comprising 23 genes in mice and humans of which 15 (*Fgf1-10*, *Fgf16-18*, *Fgf20*, and *Fgf22* in mice) encode “canonical” FGFs that function as paracrine signaling molecules and bind promiscuously to cell surface FGF receptors (FGFRs encoded by *Fgfr1-4* in mice) to elicit an extracellular signal regulated kinase (ERK)/mitogen activated kinase (MAPK) response via activating phosphorylation of ERK→phospo-ERK (pERK). Canonical FGFs are further subdivided into five subfamilies based on phylogeny and *Fgf8* subfamily members *Fgf8* and *Fgf17* are transcribed in the developing CSB in close spatiotemporal proximity posing the question of how they are coordinated (Guillemot and Zimmer, 2011; Ornitz and Itoh, 2015). Under normal conditions, GW→IG translocation is primarily attributed to Fgf8 and needs to be tightly regulated to ensure that correct numbers of RGCs leave the GW and reach the IG. Deviation above (or below) normal FGF/ERK signaling levels induces too many (or too few) RGCs to translocate with consequent disruption to CC development (Smith et al., 2006; Wang et al., 2012; Clegg et al., 2014; Gobius et al., 2016). Although Fgf17 plays a role in patterning the developing telencephalon, its importance for CC development is less clear and no CC phenotype has been reported in *Fgf17*^−/−^ embryos (Cholfin and Rubenstein, 2007, 2008). Because *Fgf8* and *Fgf17* are the principal *Fgf*s transcribed in vicinity of the GW and both activate ERK, mechanisms must exist to keep the total amount of Fgf protein (Fgf8 protein + Fgf17 protein) at the correct level to generate the correct levels of ERK activation for astroglial precursor RGCs to translocate in appropriate numbers.

Heparan sulfate (HS), the carbohydrate component of cell surface and extracellular matrix (ECM) HS proteoglycans, is a negatively charged sulfated polysaccharide that binds canonical FGFs in the ECM to regulate their movement and half-life and also functions as an obligate FGF coreceptor in FGF:FGFR:HS ternary signaling complexes on the cell surface (Guillemot and Zimmer, 2011; Balasubramanian and Zhang, 2016). HS biosynthesis is in two stages, Ext enzymes polymerize uronic acid - glucosamine disaccharides making linear [uronic acid - glucosamine]_n_ HS polymers which are then modified by the enzymatic addition (by HS sulfotransferases, HSTs) or removal (by HS sulfatases, Sulfs) of sulfate groups at specific positions on the disaccharide residues. There are four classes of HST enzymes, Hs2st, Hs3st, Hs6st, and Ndst, each adding sulfate to a specific position; for example, Hs2st only adds sulfate to the carbon atom in position 2 of uronic acid, generating 2-O HS sulfation. Although work in a variety of systems shows that HS itself can play roles both in the transmission of FGF signals through the ECM (non-cell-autonomous role) and the cellular response to FGF (cell-autonomous role), the potential for specific forms of HS sulfation to selectively regulate FGFs by regulating the physical interaction between HS and FGF proteins is much less well understood (Allen et al., 2001; Loo et al., 2001; Loo and Salmivirta, 2002; Allen and Rapraeger, 2003; Belenkaya et al., 2004; Kinnunen et al., 2005; Makarenkova et al., 2009; Yan and Lin, 2009; Yu et al., 2009; Guillemot and Zimmer, 2011; Qu et al., 2011, 2012; Toyoda et al., 2010; Christian, 2012; Zhang H et al., 2012; Ramsbottom et al., 2014; Chan et al., 2015, 2017; Balasubramanian and Zhang, 2016).

The HS code hypothesis states that different forms of HS sulfation can encode specific instructions (Turnbull et al., 2001; Kreuger et al., 2006). In this study, we discover that 2-O HS sulfation catalyzed by Hs2st functions non-cell autonomously at the developing telencephalic midline to suppress FGF/ERK signaling that drives the somal translocation of astroglial precursors required for normal CC development. We also present evidence that Hs2st plays a selective role by modulating the physical interaction between Fgf17 protein and HS and selectively suppressing Fgf17 protein levels at the CSB.

## Materials and Methods

### 

#### 

##### Animals.

All mice were bred in-house according to Home Office UK legislation and licenses approved by the University of Edinburgh Ethical Review Committees and Home Office. Embryos analyzed in this study were of either sex. Animal husbandry was in accordance with UK Animals (Scientific Procedures) Act of 1986 regulations. The *Hs2st* LacZ (*Hs2st*^−^)-null allele comprised a *LacZ* gene trap vector integrated into the *Hs2st* locus, the *Hs6st1* LacZiresPLAP (*Hs6st1*^−^)-null allele comprised a *LacZiresPLAP* gene trap vector integrated in the *Hs6st1* locus, and both were genotyped by PCR as described previously (Bullock et al., 1998; Pratt et al., 2006; Conway et al., 2011). For some *ex vivo* experiments, *Hs2st*^−/+^ mice were crossed with mice carrying the TP6.3 tau (τ)-GFP fusion transgene to generate *Hs2st*^−/−^ and *Hs2st*^+/+^ embryos with τ*GFP*^+^ axons (Pratt et al., 2000). For conditional mutagenesis, floxed *Ext1* (*Ext1*^fl^) or *Hs2st* (*Hs2st*^fl^) alleles were combined with either *Zic4*^Cre^ (septal deletion) or *Emx1*^CreER^ (cortical deletion) driver alleles (Inatani et al., 2003; Kessaris et al., 2006; Rubin et al., 2010; Stanford et al., 2010). *CreER* activity was induced at embryonic day 9.5 (E9.5) by administering tamoxifen (dissolved in corn oil using a sonicator) to pregnant dams by intraperitoneal injection (120 mg/kg dose). Lineages of cells in which Cre was active were visualized using a Rosa26R-floxed-stop-EGFP reporter allele (Sousa et al., 2009).

##### *Ex vivo* assays.

*Ex vivo* culture experiments were performed essentially as described previously (Niquille et al., 2009) Explants were cultured on nucleopore polycarbonate membranes (Whatman) floating on 1 ml of neurobasal medium (Life Technologies) supplemented with l-glutamine, glucose, and penicillin/streptomycin) at 37°C with 5% CO_2_ in a humidified incubator. Brains were dissected from embryos in oxygenated Earle's balanced salt solution (Life Technologies), embedded in low-melting-point agarose, sliced using a vibratome (Leica VTS-1000), and transferred to modified Eagle's medium (MEM; Life Technologies) with 5% fetal bovine serum for 1 h. For CC axon navigation assays, 400-μm-thick E17.5 coronal slices incorporating the CC axon tract were prepared and frontal cortex explants from τ-GFP^+^ slices were transplanted into the equivalent region in τ-GFP^−^ slices before culturing in neurobasal medium for 72 h, fixation in 4% paraformaldehyde (PFA), and GFP immunofluorescence. For glial translocation experiments, 10 mg/ml BrdU dissolved in PBS was injected intraperitoneally into pregnant dams with E14.5 litters, which were killed 1 h later and 350 μm coronal slices incorporating the CSB prepared for culture. In Fgf17 bead experiments, Affi-Gel blue gel (Bio-Rad) beads presoaked in 100 μg/ml recombinant Fgf17 protein (R&D systems) or 5 mg/ml BSA (Sigma-Aldrich) overnight at 4°C were implanted into the slice, one Fgf17 and one BSA bead on either side of the midline just below the GW, and the MEM was replaced with neurobasal medium. For the FGFi culture, MEM was replaced with neurobasal medium containing either 25 μm SU5402, 0.1% DMSO (FGFi) or 0.1% DMSO (control). Slices were cultured for 2 or 48 h, fixed in 4% PFA, and 10 μm frozen sections were prepared for immunodetection or *in situ* hybridization. Glial migration out of the VZ toward the pial surface was quantified from BrdU/Sox9 immunofluorescence micrographs by demarcating the basal edge of the VZ (easily identified by Sox9 staining) with a line and counting the number of Sox9^+^;BrdU^+^ cells that had crossed this line. This allowed us to count glial (Sox9^+^) cells that had incorporated BrdU (BrdU^+^) when they were in the VZ before the start of the culture and subsequently exited the VZ and migrated toward the midline over the 2 d culture period when the cultures were exposed to experimental substances (SU5402, DMSO, Fgf17 protein, or BSA). Four or six sections were quantified per slice moving rostrally from the most caudal section in which the GW could be identified on both sides of the section.

##### Immunodetection.

Embryonic mouse brains were removed and fixed in 4% PFA in PBS overnight at 4°C, cryoprotected in 30% sucrose in PBS, embedded in OCT, and 10 μm coronal frozen sections were cut using a cryostat (Leica). Immunohistochemistry was performed as described previously (Clegg et al., 2014). Primary antibodies: goat anti-GFP (diluted 1/250; Abcam); rabbit anti-Sox9 (1/500; Cell Signaling Technology); rat anti-L1 (1/200; Millipore); rabbit anti-GFAP (1/200 Dako); rabbit anti-Hs2st (1/50; Abcam ab103120); rabbit anti-Fgf17 (1/1000; Abcam ab187982); and rabbit anti-pErk1/2 (1/200; Cell Signaling Technology). Secondary antibodies were as follows: donkey anti-goat Alexa Fluor 488, donkey anti-rabbit Alexa Fluor 568, and goat anti-rat 568 (all used at a dilution of 1/200 and from Invitrogen). Fluorescently labeled sections were counterstained with DAPI (Invitrogen). For Hs2st and pErk1/2 antibody staining, goat anti-rabbit biotin secondary antibody (1/200; Vector Laboratories) was used and staining was visualized using a standard avidin-biotin diaminobenzidine (DAB) staining procedure. The Fgf17 immunofluorescence was performed using exactly the same protocol as described previously for Fgf8 except that the Fgf8 antibody was replaced with the Fgf17 antibody (Toyoda et al., 2010; Clegg et al., 2014). Briefly, slides were first washed in acetone for permeabilization, rabbit Fgf17 antibody was applied, and the TSA Plus Fluorescence System Kit (PerkinElmer) was used for fluorescence detection.

##### *In situ* hybridization.

*In situ* hybridization was performed on 10 μm frozen sections as described previously (Wallace and Raff, 1999) using digoxigenin-labeled riboprobes for *Slit2* and *Fgf17* (Xu et al., 1999; Erskine et al., 2000).

##### Imaging.

Fluorescence-labeled sections were imaged using either a Leica AF6000 epifluorescence microscope coupled to a Leica DFC360 digital camera or a Nikon Ti: E Inverted confocal microscope. DAB stained and *in situ* hybridized sections were imaged using a Leica DLMB microscope coupled to a Leica DFC480 color digital camera.

##### Fgf17 protein quantification.

Fgf17 fluorescence was quantified from E14.5 *Hs2st*^+/+^*;Hs6st1*^+/+^, *Hs2st*^−/−^, and *Hs6st1*^−/−^ coronal sections that had been processed for Fgf17 immunofluorescence in parallel and imaged under identical conditions in parallel using the same method as described previously for Fgf8 protein quantification (Chan et al., 2017). For each section, ImageJ was used to measure mean fluorescence intensity in a 100 × 150 μm box drawn at the CSB encompassing the Fgf17 expression domain. For each embryo, quantification was performed for three sections along the rostrocaudal axis and averaged.

##### IG Sox9^+^ cell quantification.

Quantification of Sox9^+^ cells in the IG region of E18.5 *Hs2st*^fl/fl^*;Zic4*^Cre^, *Hs2st*^+/+^*;Zic4*^Cre^, *Hs2st*^fl/fl^*;Emx1*^CreER^, and *Hs2st*^+/+^*;Emx1*^CreER^ embryos was performed as described previously (Clegg et al., 2014). A counting box measuring 200 × 200 μm was placed on images of coronal sections at the midline with the top edge at the dorsal extent of Sox9^+^ cells at the IG and the numbers of Sox9^+^ cells in the box counted. For each embryo, quantification was performed for three sections along the rostrocaudal axis and averaged.

##### Western blotting.

Western blotting was performed as described previously (Clegg et al., 2014). Primary antibodies were as follows: rabbit anti-Hs2st (1/500; Abcam ab103120) and mouse anti-β-actin (1/5000; Abcam). Secondary antibodies were as follows: goat anti-mouse Alexa Fluor 680 (Invitrogen) and goat anti-rabbit 800 (Li-Cor).

##### Ligand and carbohydrate engagement (LACE) assay.

The LACE assay was performed as described previously (Allen et al., 2001; Allen and Rapraeger, 2003; Chan et al., 2015). Briefly, frozen sections were incubated in 0.05% NABH_4_/PBS for 15 min. After several washes in PBS, sections were incubated in 0.1 m glycine at 4°C overnight. Some sections were incubated with Heparitinase I (Seikagaku) before proceeding. All Fgf and Fgfr-Fc proteins were purchased from R&D Systems. Sections were then treated with 1% BSA/TBS solution for 10 min before incubation with 3 μm recombinant mouse Fgf17 and 9 μm recombinant human Fgfr1a(IIIc)-Fc or 30 nm recombinant mouse Fgf8b and 100 nm recombinant human Fgfr3 (IIIc)-Fc at 4°C overnight. Fgf17 or Fgf8 were omitted from some assays. Fluorescent LACE signal was generated by incubation with 1/200 anti-human IgG (Fc-specific) Cy3 (Sigma-Aldrich) in 1% BSA/TBS. *Hs2st*^+/+^*;Hs6st1*^+/+^, *Hs2st*^−/−^, and *Hs6st1*^−/−^ material that had been processed for each LACE assay condition in parallel was imaged under identical conditions in parallel. For each section, ImageJ was used to measure mean fluorescence intensity in a 100 × 150 μm box drawn encompassing the CSB. Background signal was quantified from control LACE experiments from which the FGF ligand was omitted and these values were used for background subtraction. For each embryo, quantification was performed for three sections along the rostrocaudal axis and averaged.

##### Data analysis and statistics.

Results are expressed as mean ± SEM. The statistical test and sample size (*n*) for each experiment are specified in the figure legends. Statistical comparison between two groups was performed with a *t* test. Statistical comparison between more than two groups was performed with ANOVA followed by *post hoc t* test. *p* < 0.05 was considered significant.

## Results

### Hs2st protein is widely expressed in the developing cerebral cortex and at the telencephalic midline

**Figure 1.**
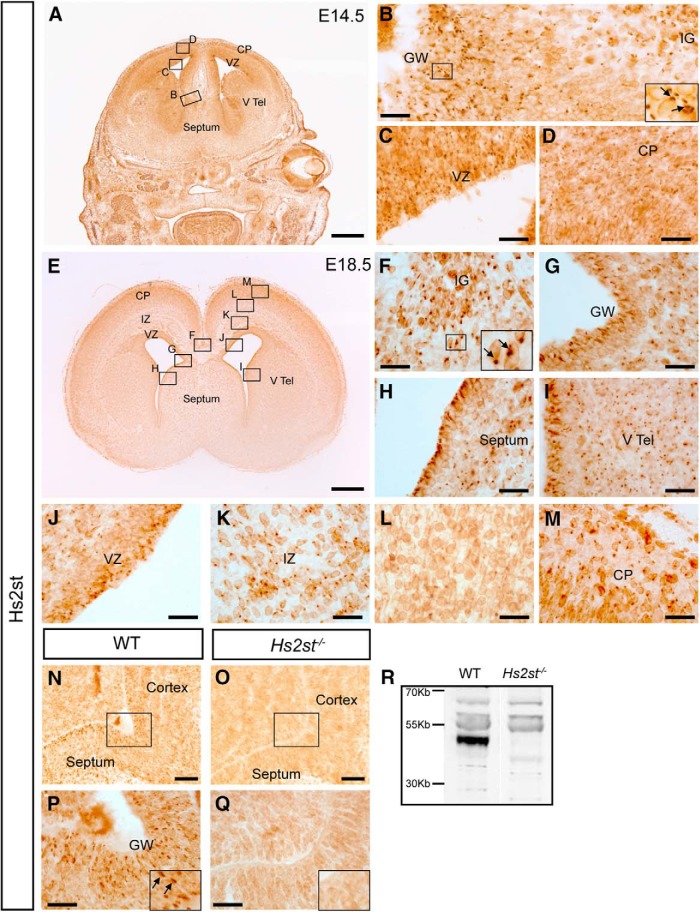
Hs2st protein is expressed in the cerebral cortex and the septum during CC formation. ***A***, Immunohistochemistry for Hs2st at E14.5. ***B***–***D***, Higher magnification showing punctate subcellular Hs2st expression (inset, ***B***) Hs2st protein is expressed at the CSB (***B***), the VZ of the cortex (***C***), and the cortical plate (***D***). ***E***, Immunohistochemistry for Hs2st at E18.5 (***F***–***M***) Hs2st protein is expressed in the IG (***F***), the GW (***G***), the septum (***H***), and the ventral telencephalon (***I***). Within the cortex, Hs2st is expressed at the VZ (***J***) and the intermediate zone (***K***); Hs2st is not strongly expressed by the middle layers of the cortex (***L***), but is expressed by the deeper layers (***M***). ***N***–***R***, Hs2st antibody specificity. The Hs2st antibody produces signal in the GW (***N***, ***P***), which is lost in *Hs2st*^−/−^ embryos (***O***, ***Q***). Western blot performed on protein extracted from whole telencephalon using Hs2st antibody revealed the predicted ∼42 kDa band in WT extracts, which is lost in *Hs2st*^−/−^ extracts (***R***). ***B***–***D*** are higher-magnification images of boxed regions indicated in ***A***. ***F***–***M*** are higher-magnification images of boxed regions indicated in ***E***. ***P*** and ***Q*** are higher-magnification images of boxed regions in ***N*** and ***O***, respectively. Insets in ***B***, ***F***, ***P*** and ***Q*** show higher magnification with arrows indicating Hs2st puncta. Scale bars: ***A***, 500 μm; ***B***–***I***, ***L***, ***M***, 50 μm; ***J***, ***K***, 100 μm.

To establish potential sites of action of Hs2st in CC development, we first examined the distribution of cells expressing Hs2st protein and contributing to developing CC structures using Hs2st immunohistochemistry at E14.5 (Fig. 1*A–D*) and E18.5 (Fig. 1*E–M*) spanning the period of CC axon tract development. Macroscopically, Hs2st protein distribution closely resembles the *Hs2st-LacZ* reporter staining previously reported, with widespread Hs2st expression in the developing cerebral cortex and at the CSB at both E14.5 and E18.5 (Fig. 1*A*,*E*, boxed areas indicate regions shown at higher magnifications in *B–D* and *F–M*) (Conway et al., 2011). Subcellularly, the Hs2st signal is punctate consistent with the expected localization of Hs2st in the Golgi apparatus (arrows point to Hs2st^+^ puncta in higher-magnification insets in Fig. 1*B*,*F*,*P*). At E14.5, there was a high density of Hs2st^+^ puncta at the CSB in the GW region where IG astroglial RGC prescursors reside (Fig. 1*B*, with boxed area shown as higher-magnification inset with arrows indicating Hs2st^+^ puncta), with the density falling toward the pial surface although Hs2st^+^ puncta were visible. There were many Hs2st^+^ puncta in the VZ of the cerebral cortex (Fig. 1*C*) and also in the cortical plate (Fig. 1*D*), indicating that many cortical progenitors and postmitotic neurons express Hs2st. At E18.5, Hs2st is expressed by many cells in the IG (Fig. 1*F*) and at the apical surface of the VZ at the GW (Fig. 1*G*), septum (Fig. 1*H*), and ventral telencephalon (Fig. 1*I*), with the number of Hs2st expressing VZ cells diminishing as distance from the ventricle increases. In the cerebral cortex, Hs2st is expressed by many cells close to the apical surface of the VZ (Fig. 1*J*). Large numbers of postmitotic cortical neurons outside of the VZ express Hs2st and, moving toward the pial surface, the density of Hs2st^+^ puncta varies with laminar position [cf. Fig. 1*K–M*, showing relatively high Hs2st^+^ puncta density in cortical layers adjacent to the pial membrane (*M*) and in the intermediate zone (*K*) and lower density in the intervening region (*L*)]. We validated the Hs2st antibody by demonstrating absence of the punctate Hs2st^+^ immunostaining in *Hs2st*^−/−^ embryonic material (cf. Fig. 1*N*,*P* and *O*,*Q*; note that the more diffuse staining persists in *Hs2st*^−/−^ tissue and we discounted this as nonspecific background) and Western blot showing that the predicted 42 kDa Hs2st protein band was present in *Hs2st*^+/+^ and absent from *Hs2st*^−/−^ telencephalic protein extracts (Fig. 1*R*). To conclude, Hs2st protein is present in developing cerebral cortex, the source of CC axons, as well as in progenitor and postmitotic cells of the CSB region constituting the environment through which midline crossing CC axons navigate. Hs2st expression analysis suggests multiple potential sites of action for 2-O HS sulfation in CC development.

### *Slit2*-expressing IG is expanded in *Hs2st*^−/−^ embryos

**Figure 2.**
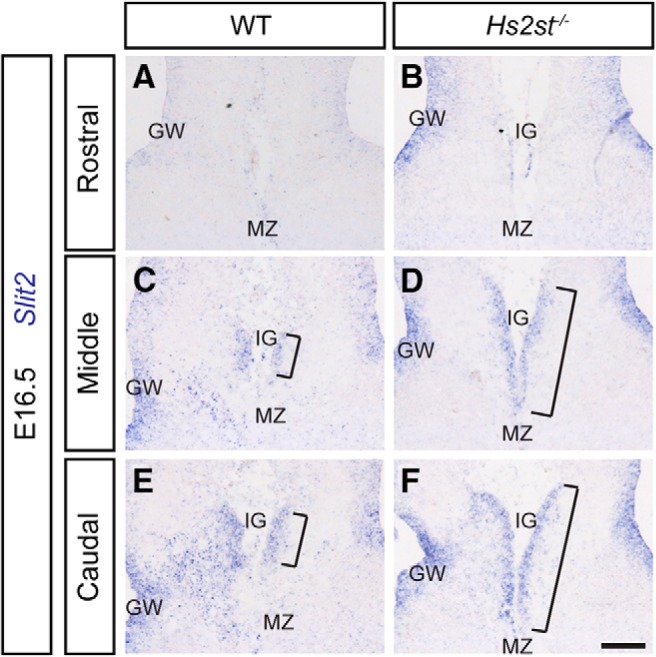
*Slit2* expression at the CSB of WT and *Hs2st*^−/−^ embryos at E16.5. ***A***, ***C***, ***E***, *In situ* hybridization for *Slit2* in WT embryos at 3 rostrocaudal positions labeling the GW and IG. ***B***, ***D***, ***F***, *In situ* hybridization for *Slit2* in *Hs2st*^−/−^ embryos at equivalent positions to ***A***, ***C***, and ***E***, respectively, showing an expanded IG. Scale bars, 100 μm in all panels.

We previously reported that increased numbers of astroglia at the pial surface of the *Hs2st*^−/−^ CSB stemmed from precocious glial translocation and found no evidence that changes in cell proliferation or death contributed to this phenotype (Conway et al., 2011; Clegg et al., 2014). To determine whether there is an expansion of the IG in *Hs2st*^−/−^ embryos, we compared the expression of *Slit2* mRNA, a marker of GW and IG glia but not MZ glia, between *Hs2st*^+/+^ and *Hs2st*^−/−^ embryos at E16.5 (Shu and Richards, 2001; Shu et al., 2003). In *Hs2st*^+/+^ embryos *Slit2*^+^ cells form a compact focus at the IG that increases in size moving caudally (Fig. 2*A*,*C*,*E*, *Slit2* expression domain at IG indicated by brackets). In *Hs2st*^−/−^ embryos, the Slit2 expression domain is greatly expanded at the pial surface along the rostrocaudal axis (Fig. 2*B*,*D*,*F*, expanded *Slit2* expression domain indicated by brackets). We conclude that an expansion of the *Slit2*^+^ IG astroglial population makes a major contribution to the *Hs2st*^−/−^ phenotype.

### Cell autonomy of HS and 2-O HS sulfation in astroglial precursor somal translocation and CC development

We next exploited conditional mutagenesis of *Hs2st* or *Ext1* to experimentally uncouple specific functions of 2-O sulfation from more general functions of HS in astroglial precursor translocation and CC development. Widespread expression of HS and 2-O HS sulfation leaves open the possibility that each regulates GW→IG astroglial precursor somal translocation cell autonomously by modulating the response to signals, non-cell autonomously by regulating the supply of signals, or both. To resolve this, we identified two *Cre* alleles, *Zic4*^Cre^ and *Emx1*^CreER^, that drive *LoxP-*mediated mutagenesis in the astroglial lineage or in their cellular environment, respectively, and used them to conditionally ablate either HS (*Ext1*^LoxP^ mutagenesis) or 2-O HS (*Hs2st*^LoxP^ mutagenesis) sulfation to test for cell-autonomous or non-cell-autonomous functions. We refer to these as “*Zic4* lineage” and “*Emx1* lineage,” respectively, and next present their characterization using a floxed-stop GFP reporter that turns on GFP expression in *Cre*-expressing cells and their descendants before describing experiments where they are used to conditionally generate loss-of-function mutations in *Ext1*^Fl^ or *Hs2st*^Fl^ alleles (Inatani et al., 2003; Kessaris et al., 2006; Rubin et al., 2010; Sousa et al., 2009; Stanford et al., 2010).

### Characterization of *Zic4* and *Emx1* lineages

**Figure 3.**
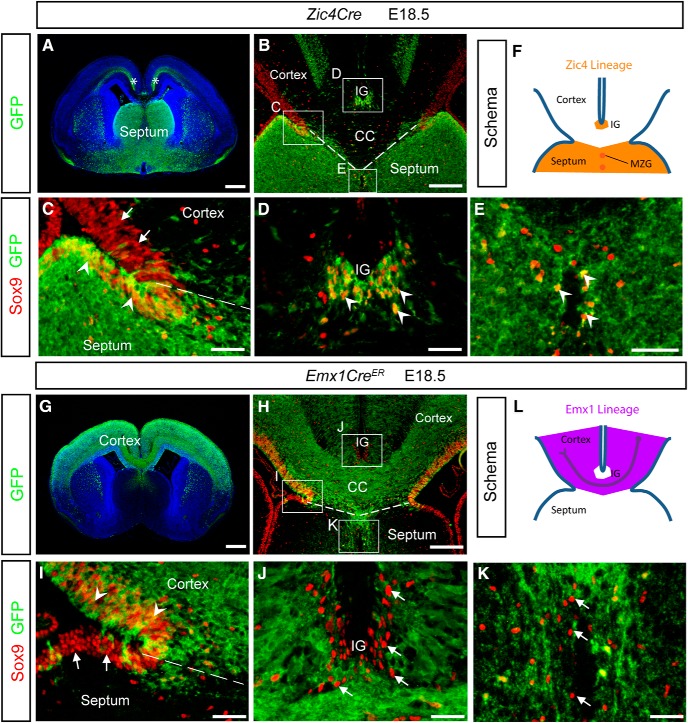
*Emx1* and *Zic4* lineage contribution at the CSB. ***A***, *Zic4*^cre^ allele combined with a lox-stop GFP reporter has been used to label cell populations at E18.5. *Zic4*^cre^ labels cells of the septum (asterisks in 3***A*** mark GFP in cortex). ***B***–***E***, *Zic4*^cre^ labels cells ventral to the CSB (dashed line, ***B***,***C***) including *Sox9* expressing cells (arrowheads, ***C***), but is not expressed by *Sox9* expressing cells dorsal to the CSB (arrows, ***C***). *Zic4*^cre^ is expressed by IG glial cells (arrowheads, ***D***) but not by surrounding cells. *Zic4*^cre^ is expressed by MZ glial cells (arrowheads, ***E***). ***F***, Schematic of the *Zic4*^cre^ expressing cell lineage. ***G***, *Emx1*^creER^ allele combined with a lox-stop GFP reporter has been used to label cell populations at E18.5. *Emx1*^creER^ labels cells of the cortex. ***H***–***K***, *Emx1*^creER^ labels cells dorsal to the CSB (dashed line, ***H***, ***I***) including *Sox9* expressing cells (arrowheads, ***I***), but is not expressed by *Sox9*-expressing cells ventral to the CSB (arrows, ***I***). *Emx1*^creER^ is not expressed by IG glial cells (arrows, ***J***). *Emx1*^creER^ is not expressed by MZ glial cells (arrows, ***K***). ***L***, Schematic of the *Emx1*-expressing cell lineage. No phenotype was detected in *Hs2st*^+/+^ *Zic4*^cre^ or *Hs2st*^+/+^ *Emx1*^creER^ embryos (*n* = 5 for each genotype). ***C***–***E*** are higher-magnification images of the indicated regions in ***B***. ***I***–***K*** are higher-magnification images of the indicated regions in ***H***. Scale bars: ***A***, ***G***, 500 μm; ***B***, ***H***, 200 μm; ***C***–***E*** and ***I***–***K***, 50 μm.

The septum is of *Zic4* lineage, as shown by strong expression of the GFP reporter (Fig. 3*A*). The GFP signal in the intermediate zone of the cerebral cortex (asterisks in Fig. 3*A*) is due to GFP^+^ thalamocortical axons that project from *Zic4*-lineage cells in the thalamus and cells of subcortical origin as described previously (Rubin et al., 2010). At the midline, GFP^+^ cells of the *Zic4* lineage are predominantly located ventral to the CSB (dashed lines in Fig. 3*B*), but there is also GFP expression in the IG (boxed area “D” in Fig. 3*B*). Sox9 is a transcription factor that marks the nuclei of RGCs in the VZ and differentiated astroglia in the IG and MZG and we previously showed that the positioning of Sox9^+^ cells is of critical importance for the development of the CC (Clegg et al., 2014). Combining GFP with Sox9 immunostaining reveals the contribution of the *Zic4* lineage to the CSB astroglial populations. There is a sharp boundary (dashed line in Fig. 3*C*) in the VZ of the CSB between Sox9^+^;GFP^+^ cells (arrowheads in Fig. 3*C*) on the septal side and Sox9^+^;GFP^−^ cells (arrows in Fig. 3*C*) on the cortical side. Virtually all the Sox9^+^ cells in the IG (Fig. 3*D*) and MZG (Fig. 3*E*) are also GFP^+^ (arrowheads in Fig. 3*D*,*E*), indicating that these cells are of the *Zic4* lineage. These data show that the *Zic4* lineage contributes Sox9^+^ cells to the septal VZ and, strikingly, is the sole source of Sox9^+^ astroglia in the IG (Fig. 3*F*).

To mark the *Emx1* lineage, tamoxifen was administered to *Emx1*^CreER^ embryos harboring the floxed-stop GFP reporter at E9.5 so that early *Emx1* expressing cerebral cortex progenitors and their descendants were rendered GFP^+^. Examination of the expression of the GFP reporter shows that, as expected, the developing cerebral cortex and CC axons are of *Emx1* lineage (Fig. 3*G*) and that, at the midline, GFP expression is predominantly located dorsal to the CSB (dashed lines in Fig. 3*H*). Higher magnification shows that there is a sharp boundary between GFP^+^ and GFP^−^ cells at the VZ of the CSB (dashed line in Fig. 3*I*). Combining Sox9 and GFP immunostaining reveals the contribution of the *Emx1* lineage to *Sox9*^+^ cells. Sox9^+^;GFP^+^ cells (arrowheads in Fig. 3*I*) populate the VZ on the cortical side of the boundary with Sox9^+^;GFP^−^ cells on the septal side (arrows in Fig. 3*I*), showing that the *Emx1* lineage contributes Sox9^+^ cells exclusively to the cortical side of the VZ. All Sox9^+^ cells in the IG (Fig. 3*J*) and MZ (Fig. 3*K*) are GFP^−^ (arrows in Fig. 3*J*,*K*), indicating that the *Emx1* lineage does not contribute Sox9^+^ cells to the IG. These data show that the *Emx1* lineage contributes Sox9^+^ cells to the cortical VZ, but no cells of this lineage contribute Sox9^+^ astroglia to the IG (schema in Fig. 3*L*).

### *Ext1* is required by both *Emx1*- and *Zic4*-lineage cells for CC development

**Figure 4.**
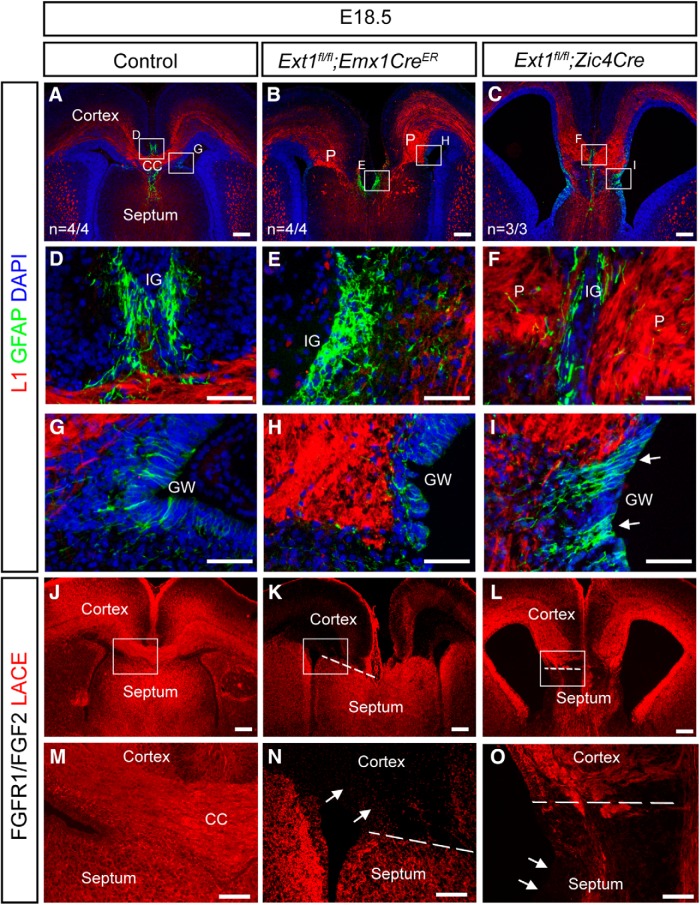
HS expression is required within both *Emx1* and *Zic4*-lineage cells for CC formation. ***A***–***I***, Immunofluorescence for L1 (red) at E18.5 labels the CC, whereas GFAP (green) labels glia. In control embryos, the U-shaped CC has formed and is flanked by glia at the IG and GW (***A***, ***D***, ***G***). In *Ext1*^fl/fl^ *Emx1*^creER^ embryos, CC axons do not cross the midline, whereas glia at the IG and GW appear largely unaffected (***B***, ***E***, ***H***). In *Ext1*^fl/fl^ *Zic4*^cre^ embryos, CC axons do not cross the midline, whereas glia appear depleted at the IG and form abnormal bundles at the GW (***C***, ***F***, ***I***). ***J***–***O***, FGFR1/FGF2 LACE assay is used to detect the presence of HS. In control embryos, the LACE signal can be seen throughout the telencephalon and is of similar intensity within both the cortex and the septum (***J***, ***M***). In *Ext1*^fl/fl^ *Emx1*^creER^ embryos, the LACE signal is significantly reduced within the cortex (***K***, ***N***). In *Ext1*^fl/fl^ *Zic4*^cre^ embryos, the LACE signal is significantly reduced within the septum (***L***, ***O***). ***D***–***I*** are higher-magnification images of the indicated boxed regions in ***A***–***C***. ***M***–***O*** are higher-magnification images of the boxed region in ***J***–***L***, respectively. Scale bars: ***A***–***C***, ***G***–***I***, 200 μm; ***D***–***F*** and ***J***–***L***, 100 μm.

To determine the cellular requirement for HS, we deleted *Ext1*, which is essential for HS synthesis, in the *Zic4* or *Emx1* lineages. In control embryos, L1 immunostaining labels axons in the U-shaped CC, whereas GFAP staining labels midline astroglial structures (Fig. 4*A*, with higher magnification of IG and GW in *D*,*G*). Removing HS from either the *Zic4* lineage (Fig. 4*B*, with higher magnification of IG and GW in Fig. 4*E*,*H*) or the *Emx1* lineage (Fig. 4*C*, with higher magnification of IG and GW in Fig. 4*F*,*I*) generates a severe CC agenesis phenotype (*Zic4*^Cre^*;Ext1*^Fl/Fl^ *n* = 4/4; *Emx1*^CreER^*;Ext1*^Fl/Fl^ *n* = 3/3). In *Emx1* conditional mutants (*Emx1*^CreER^*;Ext1*^Fl/Fl^), CC axons fail to cross the midline and form Probst bundles short of the midline (Fig. 4*B*). GFAP^+^ astroglial cells are present in the IG (Fig. 4*E*) and at the GW (Fig. 4*H*) in a pattern grossly similar to that of controls (cf. Fig. 4*D*,*G* and *E*,*H*). In *Zic4* conditional mutants (*Zic4*^Cre^*;Ext1*^Fl/Fl^), CC axons approach the midline but fail to cross (Fig. 4*C*, with higher magnification of IG and GW in *F*,*I*). Astroglial populations in *Zic4* conditional mutants are obviously disrupted, with less intense GFAP staining at the midline (cf. IG region in Fig. 4*D*,*F*) and more GFAP at the GW than in controls (arrows in Fig. 4*I*; cf. Fig. 4*G*,*I*), suggesting that, in these embryos, astroglial precursors translocate less efficiently to the IG and instead remain in the GW. The cerebral cortex of *Zic4*^Cre^*;Ext1*^Fl/Fl^ brains was thinned and the ventricles were enlarged (cf. Fig. 4*A*,*C*) and this hydrocephalus-like phenotype is intriguing because the cerebral cortex is not of the *Zic4* lineage, indicating a non-cell-autonomous mechanism by which HS regulates cerebral cortex development. The FGFR1/FGF2 LACE assay detects endogenous HS on tissue sections by forming ternary complexes with exogenously added FGF2 and FGFR1 (red LACE signal in Fig. 4*J–O*) (Allen et al., 2001; Chan et al., 2015). HS is ubiquitously expressed in both cortical and septal compartments of control telencephalon (Fig. 4*J*, higher magnification of CSB in *M*) and, as intended, HS synthesis is blocked in the cortex and cortical axons of *Emx1*^CreER^*;Ext1*^Fl/Fl^ embryos (Fig. 4*K*, CSB shown at higher magnification in *N*, with arrows indicating HS-deficient cortical region) and in the septum of *Zic4*^Cre^*;Ext1*^Fl/Fl^ embryos (Fig. 4*L*, higher magnification of CSB in *O*, with arrows indicating HS-deficient septum). Predigesting tissue sections with heparitinase eliminated the LACE signal (data not shown), confirming the specificity of this assay for detecting HS. The salient conclusions from the *Ext1* conditional mutagenesis for the current study are that HS is indispensable from both the *Zic4* and the *Emx*1 lineages for CC development and that removing HS from the *Zic4* lineage inhibits *Zic4* lineage astroglia from reaching the IG region.

### *Hs2st* is required by *Emx1*-lineage but not *Zic4*-lineage cells for CC development

**Figure 5.**
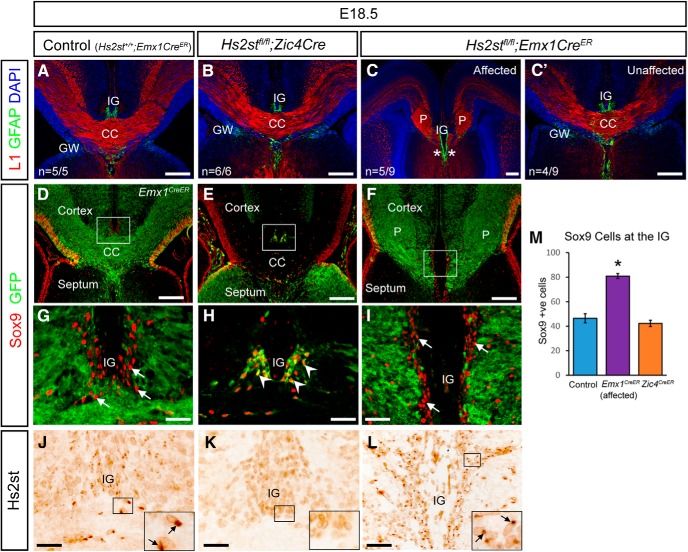
*Hs2st* expression is required within *Emx1*-lineage cells but not *Zic4*-lineage cells for CC formation. ***A***–***C***, Immunofluorescence for L1 and GFAP at E18.5. In control embryos, the U-shaped CC has formed and the IG can be observed above the CC (***A***). In *Hs2st*^fl/fl^ *Zic4*^cre^ embryos, the CC and IG form normally (***B***). In approximately half of *Hs2st*^fl/fl^;*Emx1*^creER^ embryos, the CC fails to form, IG glia also extend ventrally (asterisks, ***C***). In the remaining *Hs2st*^fl/fl^;*Emx1*^creER^ embryos, the CC forms normally (***C′***). ***D***–***I***, Immunofluorescence for *Sox9* labels progenitor cells at the VZ and mature glia at the IG, GFP labels cells in which cre is active. ***D***, ***G***, In control (*Hs2st*^+/+^;*Emx1*^creER^) embryos, IG glia do not express GFP. ***E***, ***H***, In *Hs2st*^fl/fl^;*Zic4*^cre^ embryos, IG glia do express GFP and adopt their normal position. ***F***, ***I***, In *Hs2st*^fl/fl^;*Emx1*^creER^ embryos, GFP is expressed by cortical neurons and axons but not by abnormally positioned IG glia. ***J***–***L***, Immunohistochemistry for Hs2st shows expression of Hs2st in the IG. ***J***, In control embryos, punctate Hs2st staining can be seen within IG cells. In *Hs2st*^fl/fl^ *Zic4*^cre^ embryos, Hs2st is not expressed by IG glia (***K***). In *Hs2st*^fl/fl^;*Emx1*^creER^ embryos, Hs2st is expressed by displaced glial cells (***L***). Hs2st immunohistochemistry in ***J***–***L*** was performed on adjacent tissue sections to those in ***D***–***I***. ***M***, Quantification of Sox9 expressing cell number at the IG in control (blue bar, *n* = 4 embryos, *2 Hs2st*^+/+^;*Zic4*^cre^+ *2 Hs2st*^+/+^;*Emx1*^creER^), affected *Hs2st*^fl/fl^;*Emx1*^creER^ (orange bar, *n* = 4 embryos), and *Hs2st*^fl/fl^;*Zic4*^cre^ (purple bar, *n* = 3 embryos). Sox9^+^ numbers are significantly increased compared with control in *Hs2st*^fl/fl^;*Emx1*^creER^ embryos (*p < 0.05; *F*_(2,7)_ = 42.16, *p* = 0.00013, ANOVA, *post hoc t* tests: control vs *Hs2st*^fl/fl^;*Emx1*^creER^; *t*_(4)_ = −8.08, *p* = 0.0013, *t* test; and control vs *Hs2st*^fl/fl^;*Zic4*^cre^; *t*_(5)_ = 0.92, *p* = 0.40, *t* test). ***G***–***I*** are higher-magnification images of boxed regions in ***D***–***F***, respectively. Insets in ***J***–***L*** are higher-magnification images of boxed region shown on each image. Scale bars: ***A***–***F***, 200 μm; ***G***–***L***, 50 μm.

Having established that both *Zic4* and *Emx1* lineages need to synthesize HS for normal CC development, we next investigated whether 2-O HS sulfation of the HS is required in either lineage. Hs2st is the sole enzyme capable of imparting 2-O HS sulfation onto HS, so to determine the cellular requirement for 2-O HS sulfation, we deleted *Hs2st* in the *Zic4* or *Emx1* lineages. Control *Hs2st*^+/+^ genotypes (*Hs2st*^+/+^*;Emx1*^CreER^ and *Hs2st*^+/+^*;Zic4*^Cre^) displayed neither CC agenesis nor midline astroglial disorganization; the control embryo shown in Figure 5 (*A*,*D*,*G*,*J*; *D* and *G* are reproduced from Fig. 3*H*,*J*) is of the *Hs2st*^+/+^*;Emx1*^CreER^ genotype. The CC and the midline astroglial structures form normally in *Hs2st*^fl/fl^*;Zic4*^Cre^ conditional mutants and the organization of L1^+^ axons and GFAP^+^ astroglia are indistinguishable from control embryos (6/6 embryos) (cf. Fig. 5*B*,*A*). The organization of GFP^+^ *Zic4*-lineage cells is the same in *Hs2st*^fl/fl^*;Zic4*^Cre^ embryos as in *Hs2st*^+/+^*;Zic4*^Cre^ embryos (cf. Figs. 5*E*,*H* and 3*B*,*D*) and IG Sox9^+^ cell counts confirm that the numbers of Sox9^+^ cells in the IG are not significantly different from control *Hs2st*^+/+^ embryos (Fig. 5*M*, cf. blue and orange bars), indicating no cell-autonomous requirement for *Hs2st* in the *Zic4*-lineage Sox9^+^ IG astroglia. To exclude the possibility of a compensatory mechanism by which the *Hs2st*^fl/fl^*;Zic4*^Cre^ IG is populated by *Hs2st*^+/+^ cells from a different lineage, we performed Hs2st immunohistochemistry and confirmed that Hs2st expression is indeed absent from all cells in the IG (Fig. 5*K*, note this is an adjacent section from the same embryo to the one shown in *H*). In *Hs2st*^fl/fl^*;Emx1*^CreER^ embryos, the CC fails to form in ∼50% of cases and embryos either had a severe phenotype (Fig. 5*C*, 5/9 embryos) or appeared completely unaffected (Fig. 5*C′*, 4/9 embryos). CC axons form Probst bundles on either side of the telencephalic midline, and the GFAP^+^ IG is expanded (asterisks in Fig. 5*C*). The anatomy and incomplete penetrance of the CC phenotype in *Hs2st*^fl/fl^*;Emx1*^CreER^ embryos closely resemble constitutive null *Hs2st*^−/−^ embryos, indicating that *Hs2st* function within the *Emx1* lineage is sufficient for normal CC development (Conway et al., 2011; Clegg et al., 2014). As in control *Hs2st*^+/+^*;Emx1*^CreER^ embryos (Fig. 5*D*,*G*), the GFP and Sox9 signals did not overlap in the IG region of control or *Hs2st*^fl/fl^*;Emx1*^CreER^ embryos (Fig. 5*F*, boxed area shown at higher magnification in *I*) and the Sox9^+^ cells in the IG of control embryos and the expanded IG of *Hs2st*^fl/fl^;*Emx1*^CreER^ embryos were GFP^−^ (arrows in Fig. 5*I* indicate Sox9^+^;GFP^−^ cells). Counts of Sox9^+^ cells confirmed a significant increase in the IG of affected *Hs2st*^fl/fl^*;Emx1*^CreER^ embryos compared with controls (Fig. 5*M*, cf. blue and purple bars). Immunostaining for Hs2st on adjacent sections confirmed that IG cells in *Hs2st*^fl/fl^*;Emx1*^CreER^ embryos retain Hs2st protein expression (Fig. 5*L*). Because Sox9^+^ IG astroglia do not belong to the *Emx1* lineage, their ectopic position in *Hs2st*^fl/fl^*;Emx1*^CreER^ embryos despite retaining *Hs2st* function allows us to conclude a non-cell-autonomous requirement for Hs2st in the translocation of astroglial precursors to the IG.

The salient conclusions from these conditional mutagenesis experiments are that, although the *Zic4*-lineage astroglia do require *Ext1* to form midline astroglial structures, they do not require *Hs2st*, strongly suggesting that, whereas these *Zic4*-lineage cells require HS on their cell surface to respond to translocation signals, there is no need for the HS to be 2-O sulfated. In contrast, *Hs2st* is absolutely required in the surrounding *Emx1*-lineage cells, indicating a non-cell-autonomous mechanism by which 2-O HS sulfation controls the transmission of translocation signals to the *Zic4*-lineage astroglial precursors.

### *Hs2st* is not required cell autonomously by CC axons to navigate the midline

**Figure 6.**
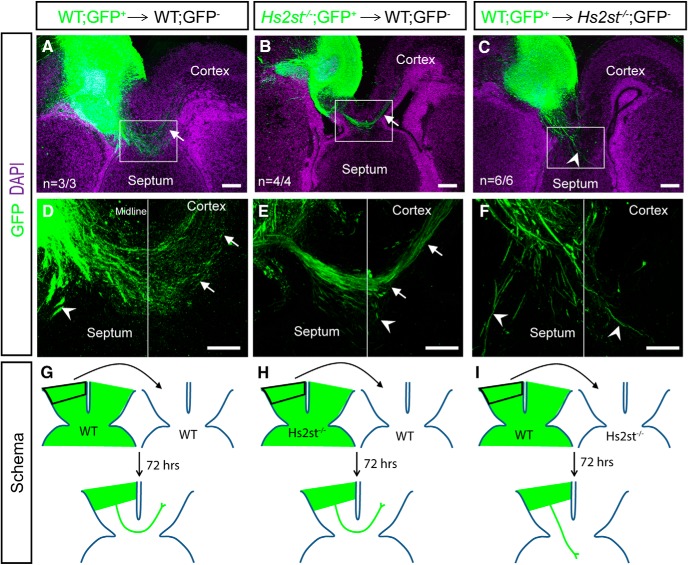
*Hs2st* is not required by CC axons to cross the telencephalic midline. ***A***, ***D***, After homotypic transplantation of E17.5 cortical explants from GFP^+^ control tissue into the cortex of GFP^−^ control brain slices, GFP^+^ CC axons are able to project across the midline (arrows, ***D***). ***B***, ***E***, After transplantation of GFP^+^ *Hs2st*^−/−^ cortical explants into GFP^−^ control brain slices, GFP^+^ CC axons are able to project across the midline (arrows, ***E***). ***C***, ***F***, After transplantation of cortical explants from GFP^+^ control tissue into the cortex of GFP^−^ *Hs2st*^−/−^ brain slices, GFP^+^ CC axons are unable to project across the midline and invade the septum. Arrowheads indicate axons navigating into the septum in all conditions. ***G***–***I***, Schematic of transplant experiments shown in ***A***–***C***. ***D***–***F*** are higher-magnification images of the boxed region in ***A***–***C***, respectively. Scale bars, 200 μm in all panels.

The conditional mutagenesis experiments showed that Hs2st has a non-cell-autonomous role in GW→IG somal translocation, but because Hs2st is expressed throughout the cerebral cortex, did not resolve whether there is an additional cell autonomous requirement in CC axon navigation. To solve this, we performed *ex vivo* transplantation experiments in which cerebral cortical tissue from transgenic mice ubiquitously expressing τGFP, which efficiently labels axons of τGFP^+^ cells, was transplanted into τGFP^−^ telencephalic slices containing the CC axon pathway and CSB structures (Pratt et al., 2000; Niquille et al., 2009). When WT E17.5 τGFP^+^ cortical explants are transplanted into age-matched τGFP^−^ WT cortical slices, τGFP^+^ axons extend across the telencephalic midline, forming the characteristic U shape of the CC and reaching the cortex of the opposite hemisphere (*n* = 3/3 cultures, arrows in Fig. 6*A*,*D* point to crossing axons). When *Hs2st*^−/−^ τGFP^+^ cortical explants are transplanted into τGFP^−^ WT slices, axons are able to cross the midline to reach the opposite hemisphere in a manner indistinguishable from that seen in the WT→WT transplants (*n* = 4/4 cultures, arrows in Fig. 6*B*,*E* point to crossing axons). In contrast, when τGFP^+^ WT cortical explants are transplanted into τGFP^−^ *Hs2st*^−/−^ slices, axons are unable to reach the opposite cortical hemisphere and instead remain within the cingulate cortex or invade the septum (*n* = 6/6 cultures, Fig. 6 *C*,*F*, arrowheads point to axons growing into the septum), resembling the *in vivo* CC phenotype observed in *Hs2st*^−/−^ embryos (Conway et al., 2011; Clegg et al., 2014). In all cultures, a few axons grew into the septum (arrowheads in Fig. 6*D–F*). Schematics summarizing these experiments are shown in Figure 6, *G–I*. These data show that 2-O HS sulfation is not required cell autonomously by CC projection neurons for axon guidance across the midline, strongly suggesting that disorganization of midline guidepost astroglial cells is the primary cause of the *Hs2st*^−/−^ CC agenesis phenotype.

### Abnormally high FGF/ERK signaling causes the *Hs2st*^−/−^ precocious astroglial translocation phenotype

**Figure 7.**
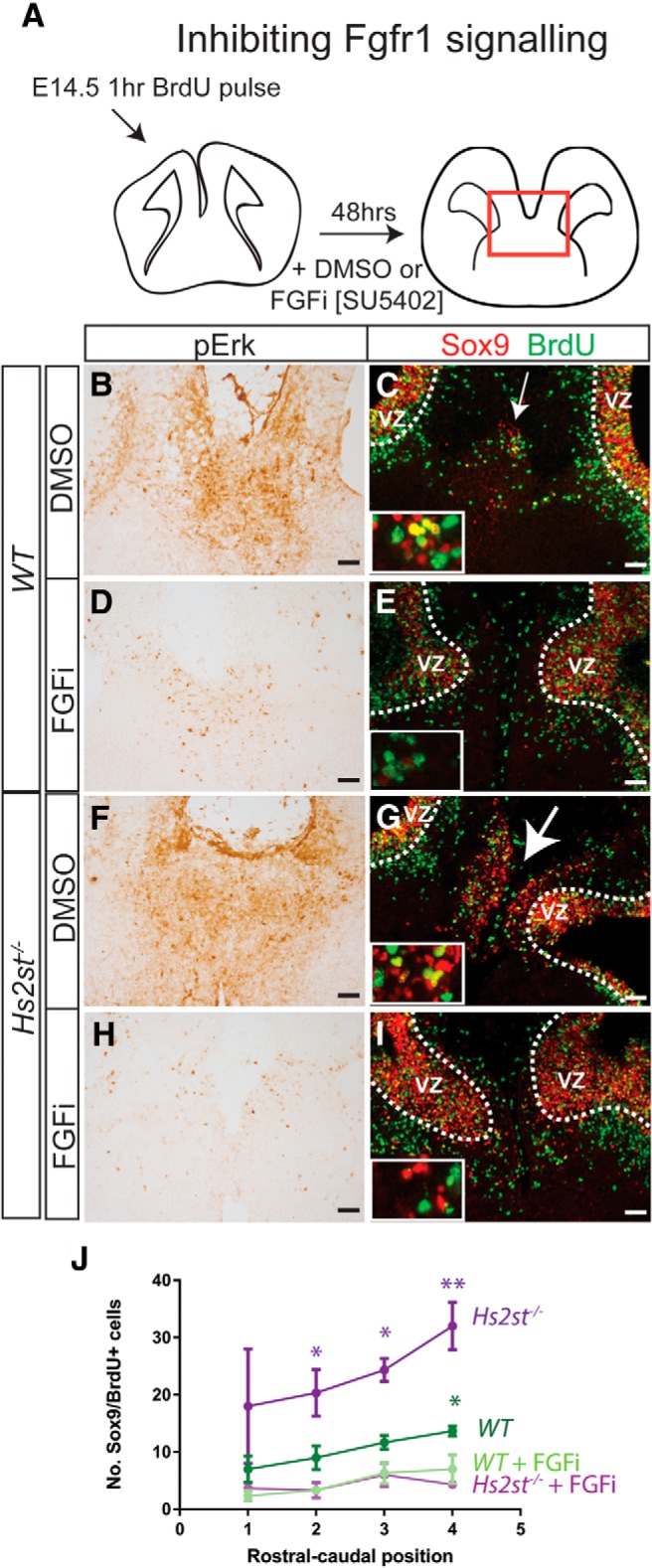
Hyperactive Fgf signaling causes precocious glia translocation in *Hs2st*^−/−^ CSB. ***A***, Experimental outline of *Hs2st*^−/−^ phenotypic rescue experiment. Pregnant females were injected at E14.5 with a BrdU pulse and CSB slices collected after 1 h and cultured for 48 h. ***B***–***I***, WT or *Hs2st*^−/−^ CSB slices were cultured in the presence of either SU5402 (FGFi) or DMSO (untreated vehicle control). ***B***, ***D***, ***F***, ***H***, pErk immunohistochemistry showing that FGFi treatment reduces Fgf/ERK signaling. (***C***,***E***,***G***,***I***) Immunofluorescence for BrdU and Sox9 in WT (***C***,***E***) and *Hs2st*^−/−^ (***G***,***I***) slices treated with FGFi (***E***,***I***) or untreated (***C***,***G***). Curved dotted line demarcates the basal edge of the VZ, arrows in ***C*** and ***G*** point to accumulations of BrdU/Sox9^+^ cells at the midline (arrow size corresponds to cell number), with higher magnification insets showing Sox9/BrdU^+^ (yellow) double-labeled cells in IG region. ***J***, Quantification of Sox9/BrdU^+^ double-labeled cells in WT or *Hs2st*^−/−^ CSB slice cultures treated with FGFi or untreated (*n* = 3 embryos for each condition). For both genotypes, FGFi treatment significantly reduced the number of Sox9/BrdU^+^ cells that exit the VZ and moved toward the IG at one or more rostrocaudal position [significant differences due to FGFi treatment within each genotype indicated on graph as *p < 0.05, **p < 0.01; *F*_(3,32)_ = 31.00, *p* = 0.0000000014, two-way ANOVA, followed by *t* test with Sidak's correction for multiple comparisons at each positon along the rostrocaudal axis. WT FGFi vs WT untreated: position 1 (*t*_(16)_ = 1.67, *p* = 0.24, *t* test); position 2 (*t*_(16)_ = 2.37, *p* = 0.11, *t* test); position 3 (*t*_(16)_ = 2.25, *p* = 0.15, *t* test); and position 4 (*t*_(16)_ = 2.81, *p* = 0.050, *t* test). *Hs2st*^−/−^ FGFi vs *Hs2st*^−/−^ untreated: position 1 (*t*_(16)_ = 2.38, *p* = 0.11, *t* test); position 2 (*t*_(16)_ = 2.83, *p* = 0.048, *t* test); position 3 (*t*_(16)_ = 3.05, *p* = 0.030, *t* test); and position 4 (*t*_(16)_ = 4.60, *p* = 0.0012, *t* test]. Scale bars in ***B***–***I***, 100 μm.

We previously reported a correlation between hyperactive ERK signaling at the CSB and precocious somal translocation of astroglia to the midline in *Hs2st*^−/−^ embryos, but we did not formally establish that this stemmed from hyperactive FGF/ERK signaling (Clegg et al., 2014; Chan et al., 2017). To address this, we used an *ex vivo* assay in which coronal WT or *Hs2st*^−/−^ telencephalic slices incorporating the CSB were cultured on floating membranes for long enough to allow somal translocation to the midline and attempted to rescue the *Hs2st*^−/−^ phenotype by pharmacological abrogation of FGF/ERK signaling. WT or *Hs2st*^−/−^ E14.5 slices were cultured in the presence of the Fgfr1 inhibitor SU5402 dissolved in DMSO (FGFi treatment) to inhibit FGF/ERK signaling or in DMSO alone (untreated control) for 48 h (Fig. 7*A*). To aid subsequent identification of translocating cells, a subpopulation RGCs undergoing S-phase in the VZ at E14.5 were labeled just before culturing with a single pulse of BrdU. Immunohistochemistry for pErk (brown stain in Fig. 7*B*,*D*,*F*,*H*) confirms inhibition of FGF/ERK signaling in both FGFi treated WT and *Hs2st*^−/−^ cultures (Fig. 7*D*,*H*) compared with untreated cultures (Fig. 7*B*,*F*), showing that FGF signaling through Fgfr1 accounts for ERK phosphorylation in both genotypes. This demonstrates that ERK hyperactivation in *Hs2st*^−/−^ embryos does not stem from an FGF-independent mechanism for ERK activation (Clegg et al., 2014; Chan et al., 2017). After 48 h, some Sox9^+^ cells (red) had left the VZ and translocated to the midline in untreated WT cultures (arrow in Fig. 7*C*), with many more populating the midline in untreated *Hs2st*^−/−^ cultures (arrow in Fig. 7*G*), validating that our *ex vivo* assay replicates the *in vivo Hs2st*^−/−^ phenotype. Consistent with our hypothesis, FGFi treatment of both WT and *Hs2st*^−/−^ cultures resulted in a large decrease in Sox9^+^ cells reaching the midline (cf. Fig. 7*E*,*I* and *C*,*G*). We quantified glial translocation by counting the numbers of Sox9^+^ cells born in the VZ at E14.5 (Sox9^+^;BrdU^+^ cells, yellow; inset in Fig. 7*C*,*E*,*G*,*I* shows higher magnification) that had exited the VZ toward the midline (VZ demarcated by dotted line in Fig. 7*C*,*E*,*G*,*I*) after 2 d in culture. Counts of BrdU^+^;Sox9^+^ cells showed that glial translocation was significantly greater in *Hs2st*^−/−^ compared with WT cultures along the rostrocaudal axis (dark purple and green lines in Fig. 7*J*) and in both cases was almost completely suppressed by FGFi treatment (pale purple and green lines in Fig. 7*J*).

We conclude that the precocious glial translocation phenotype in H*s2st*^−/−^ embryos is caused by hyperactive FGF/ERK signaling from E14.5 on. Together with our *Hs2st* conditional mutagenesis experiments demonstrating a non-cell-autonomous role for *Hs2st* in astroglial precursor translocation, we hypothesize that Hs2st normally suppresses the supply of FGF proteins to translocation competent astroglial precursors in the GW.

### *Hs2st* suppresses Fgf17 protein levels

**Figure 8.**
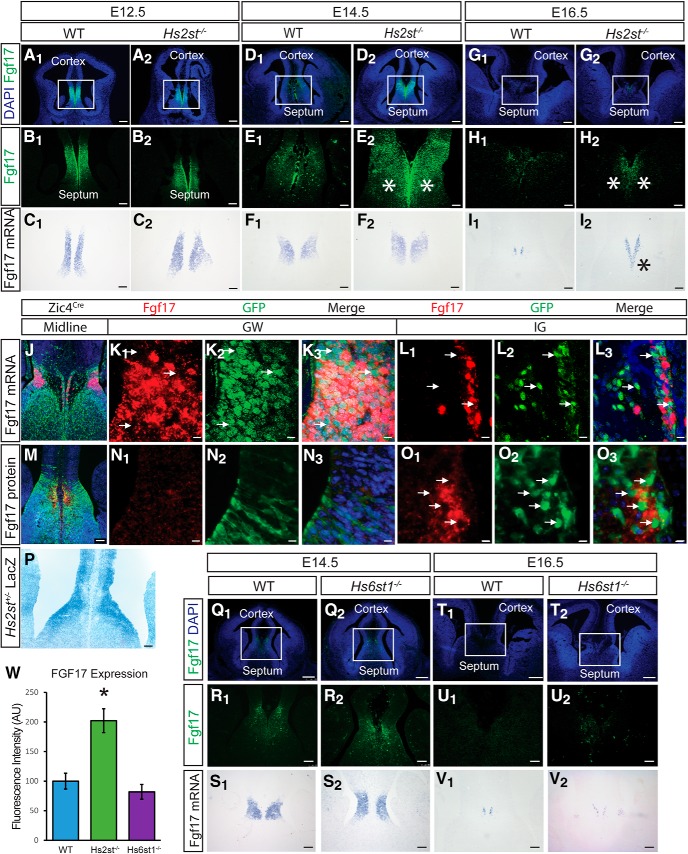
Expression of Fgf17 during CSB development in WT and *Hs2st*^−/−^ and *Hs6st1*^−/−^ embryos. ***A***–***C***, Fgf17 protein (***A,B***) and mRNA (***C***) expression at the E12.5 CSB of WT and *Hs2st*^−/−^ embryos. Fgf17 protein is expressed across the CSB in both WT and *Hs2st*^−/−^ embryos, with no obvious change in intensity or domain of expression. *Fgf17* mRNA expression overlaps well with the protein expression domain and is similar between WT and *Hs2st*^−/−^. ***D***–***F***, Fgf17 protein (***D, E***) and mRNA (***F***) expression at the E14.5 CSB of WT and *Hs2st*^−/−^ embryos. Fgf17 protein is expressed at low levels at the CSB of WT embryos. In *Hs2st*^−/−^ embryos, the protein expression domain expands across the CSB (asterisks in ***E2***). *Fgf17* mRNA is unchanged at the CSB between WT and *Hs2st*^−/−^ embryos. ***G***–***I***, Fgf17 protein (***G, H***) and mRNA (***I***) expression at the E16.5 CSB of WT and *Hs2st*^−/−^ embryos. Fgf17 protein is expressed at low levels at the CSB of WT embryos (***H1***). In *Hs2st*^−/−^ embryos, the protein expression domain expands (asterisks, ***H2***). There is a concurrent increase in *Fgf17* mRNA (asterisk, ***I2***). ***J***–***O***, Fgf17 mRNA (***J***, ***K1***– ***K3***, ***L1***–***L3***) and Fgf17 protein (***M***, ***N1***–***N3***, ***O1***–***O3***) expression (red) relative to GFP^+^ *Zic4*-lineage cells (indicated with white arrows) at the GW (***K1***–***K3***, ***N1***–***N3***) and IG (***L1***–***L3***, ***O1***–***O3***) of WT E14.5 embryos. ***P***, E14.5 expression of Hs2st by LacZ staining. Hs2st is expressed most highly at the VZ, with decreasing expression toward the pial surface. ***Q***–***V***, Fgf17 protein (***Q, R***) and mRNA (***S***) expression at the E14.5 CSB of WT and *Hs6st1*^−/−^ embryos. Fgf17 protein is expressed at low levels at the CSB of WT embryos (***Q1***, ***R1***). In *Hs6st1*^−/−^ embryos, the protein expression domain is similar to WT (***Q2***, ***R2***). *Fgf17* mRNA expression is unchanged between WT and *Hs6st1*^−/−^ embryos (***S1***, ***S2***). ***T***–***V***, Fgf17 protein (***T***, ***U***) and mRNA (***V***) expression at the E16.5 CSB of WT and *Hs6st1*^−/−^ embryos. Fgf17 protein is expressed at very low levels at the CSB of both WT (***T1***, ***U1***) and *Hs6st1*^−/−^ (***T2***, ***U2***) embryos. *Fgf17* mRNA expression is unchanged between WT (***V1***) and *Hs6st1*^−/−^ (***V2***) embryos. ***W***, Quantification of Fgf17 immunofluorescence signal at E14.5 CSB in WT (blue bar, *n* = 3 embryos), *Hs2st*^−/−^ (green bar, *n* = 3 embryos), and *Hs6st1*^−/−^ (purple bar, *n* = 3 embryos). Fgf17 protein level is significantly increased compared with WT in *Hs2st*^−/−^ embryos (*p < 0.05 on graph, *F*_(2,9)_ = 13.83, *p* = 0.0018, ANOVA, *post hoc t* tests: WT vs *Hs2st*^−/−^, *t*_(4)_ = −4.22, *p* = 0.014, *t* test; and WT vs *Hs6st1*^−/−^, *t*_(6)_ = −0.98, *p* = 0.36, *t* test). Boxed areas in ***A***, ***D***, ***G***, ***Q***, and ***T*** are shown at higher magnification in ***B***, ***E***, ***H***, ***R***, and ***U***, respectively. Scale bars: ***A***, ***D***, ***G***, ***Q***, ***T***, 200 μm; ***B***, ***C***, ***E***, ***F***, ***H***, ***I***, ***R***, ***S***, ***U***, ***V***, ***J***, ***M***, ***P***, 100 μm; ***K***, ***L***, ***N***, ***O***, 10 μm.

We next sought to identify an FGF protein that is targeted by *Hs2st*. Despite its well known role in CC development, Fgf8 protein levels are not significantly increased at the CSB of *Hs2st*^−/−^ embryos, forcing us to consider other FGFs (Clegg et al., 2014; Chan et al., 2017). A promising candidate is *Fgf17*, a member of the *Fgf8* subfamily transcribed at the CSB in a similar pattern to *Fgf8* (Cholfin and Rubenstein, 2008; Zhang X et al., 2012). Fgf17 is a canonical FGF that binds to HS, so it is potentially regulated via its interaction with HS and is known to play a role in patterning the telencephalon, although its role in CC development has not been fully characterized (Cholfin and Rubenstein, 2007; Hoch et al., 2015; Li and Kusche-Gullberg, 2016). We hypothesized that Hs2st normally suppresses Fgf17 protein and predicted that Fgf17 protein levels would be increased at the *Hs2st*^−/−^ CSB. We compared the expression of Fgf17 protein in the developing CSB of WT and *Hs2st*^−/−^ embryos at three developmental stages, E12.5, E14.5, and E16.5, spanning the interval of midline glial translocation. At E12.5, telencephalic Fgf17 protein is restricted to the CSB region with no obvious difference between WT and *Hs2st*^−/−^ (cf. Fig. 8*A1*,*B1* and *A2*,*B2*). By E14.5, there is an expanded Fgf17 protein domain at the CSB of *Hs2st*^−/−^ embryos (cf. Fig. 8*D1*,*E1* and *D2*,*E2*; *E2 marks the expanded Fgf17 protein domain). Quantification of Fgf17 immunofluorescence shows a significant ∼2-fold increase in Fgf17 protein levels in this region of *Hs2st*^−/−^ CSB (Fig. 8*W*, cf. blue and green bars). At E16.5, Fgf17 protein is much closer to detection threshold than at the earlier stages in both genotypes (Fig. 8*G1*,*G2*,*H1*,*H2*) although the increased protein spread in the mutant persists (* in Fig. 8*H*2), indicating that the *Hs2st*^−/−^ CSB is exposed to a prolonged overdose of Fgf17 protein spanning E14.5–E16.5. We next examined *Fgf17* mRNA at the CSB to determine whether the increase in Fgf17 protein in *Hs2st*^−/−^ CSB was underpinned by altered *Fgf17* gene expression. There was no evidence for this at E12.5 or E14.5, when the *Fgf17* mRNA expression pattern remained similar between *Hs2st*^+/+^ and *Hs2st*^−/−^ embryos (cf. Fig. 8*C1*,*C2* and *F1*,*F2*); however, the expression domain of *Fgf17* mRNA is increased in E16.5 *Hs2st*^−/−^ CSB (cf. Fig. 8*I1*and *I2*_,_ * in *I2* marks expanded *Fgf17* mRNA domain). This subsequent increase in *Fgf17* mRNA in the E16.5 *Hs2st*^−/−^ CSB indicates that the *Hs2st*^−/−^ phenotype has a transcriptional component or that there are more cells expressing *Fgf17* mRNA in the expanded *Hs2st*^−/−^ IG, although this cannot be the primary event because it is not apparent at E14.5, the stage at which we previously showed that precocious astroglial precursor translocation was well under way in *Hs2st*^−/−^ embryos (Clegg et al., 2014).

Mosaic analysis (Fig. 5) indicated that *Hs2st* function in the *Emx1* lineage negatively regulates a signal promoting GW→IG translocation of *Zic4*-lineage glial cells by a non-cell-autonomous mechanism and Fgf17 expression analysis (Fig. 8) makes Fgf17 a strong candidate for the signal. Based on this, we hypothesized that Fgf17 is expressed in cells surrounding the *Zic4*-lineage cells and performed detection of *Fgf17* mRNA or protein in E14.5 WT embryos in which the *Zic4* lineage is labeled GFP^+^. *Fgf17* mRNA is expressed at the GW and the IG (Fig. 8*J*) and higher-power magnification shows that, in the VZ, GFP^+^ cells express little if any *Fgf17* mRNA and, conversely, cells expressing the highest levels of *Fgf17* mRNA are GFP^−^ (Fig. 8*K1–K3*, arrows indicate GFP^+^ cell location). This complementarity between Fgf17 mRNA-expressing and *Zic4*-lineage cells is preserved at the IG (Fig. 8*L1–L3*, arrows indicate GFP^+^ cell location). Fgf17 protein predominates at the IG (Fig. 8*M*) and higher-power magnification shows that, although Fgf17 protein is barely detectable at the GW (Fig. 8*N1–N3*), there are a number of much more highly Fgf17-expressing cells at the IG and these cells are GFP^−^, confirming that they do not belong to the *Zic4* lineage (Fig. 8*O1–O3*, arrows indicate GFP^+^ cell location). Interestingly, although cells in the GW and IG express comparable levels of *Fgf17* mRNA (cf. Fig. 8*K1*,*L1*), the expression of Fgf17 protein is much higher in the IG (cf. Fig. 8*N1*,*O1*), suggesting a posttranscriptional repression selectively at the GW. Our identification of Hs2st as a repressor of Fgf17 protein levels at this stage makes Hs2st a strong candidate; indeed, closer examination of *Hs2st* expression using the *Hs2st-LacZ* reporter shows that Hs2st is expressed in a GW^High^-IG^Low^ pattern (Fig. 8*P*, also apparent in the Hs2st immunohistochemistry; Fig. 1*B*) complementary to the GW^Low^-IG^High^ Fgf17 protein distribution. Together, these data bolster the idea that Hs2st acts to suppress Fgf17 protein supply to *Zic4*-lineage cells via a posttranscriptional mechanism.

We conclude that Hs2st primarily suppresses the level and spread of Fgf17 protein emanating from the *Emx1* lineage in the CSB.

### *Hs6st1* does not affect Fgf17 protein levels

We next addressed whether the ability of 2-O HS sulfation to suppress Fgf17 protein levels *in vivo* represented a specific function of Hs2st or was redundant with other HSTs. We chose to examine Hs6st1, an HST that catalyzes 6-O HS sulfation, because we have previously shown that *Hs6st1* (but not *Hs2st*) suppresses levels of the closely related Fgf8 protein at the CSB *in vivo* (Clegg et al., 2014; Chan et al., 2017). However, we were unable to detect increased expression of Fgf17 protein (cf. Fig. 8*Q1*,*R1* and *Q2*,*R2* and *T1*,*U1* and *T2*,*U2*) or *Fgf17* mRNA (cf. Fig. 8*S1*,*S2* and *V1*,*V2*) in *Hs6st1*^−/−^ compared with WT CSB at either E14.5 or E16.5. Quantification of Fgf17 immunofluorescence shows unchanged Fgf17 protein levels in this region of *Hs6st1*^−/−^ CSB (Fig. 8*W*, cf. blue and purple bars). These data demonstrate that the negative relationship between *Hs2st* and *Fgf17* is selective *in vivo* because it does not apply to *Hs6st1*.

### Exogenously applied Fgf17 phenocopies the *Hs2st*^−/−^ astroglial translocation phenotype

**Figure 9.**
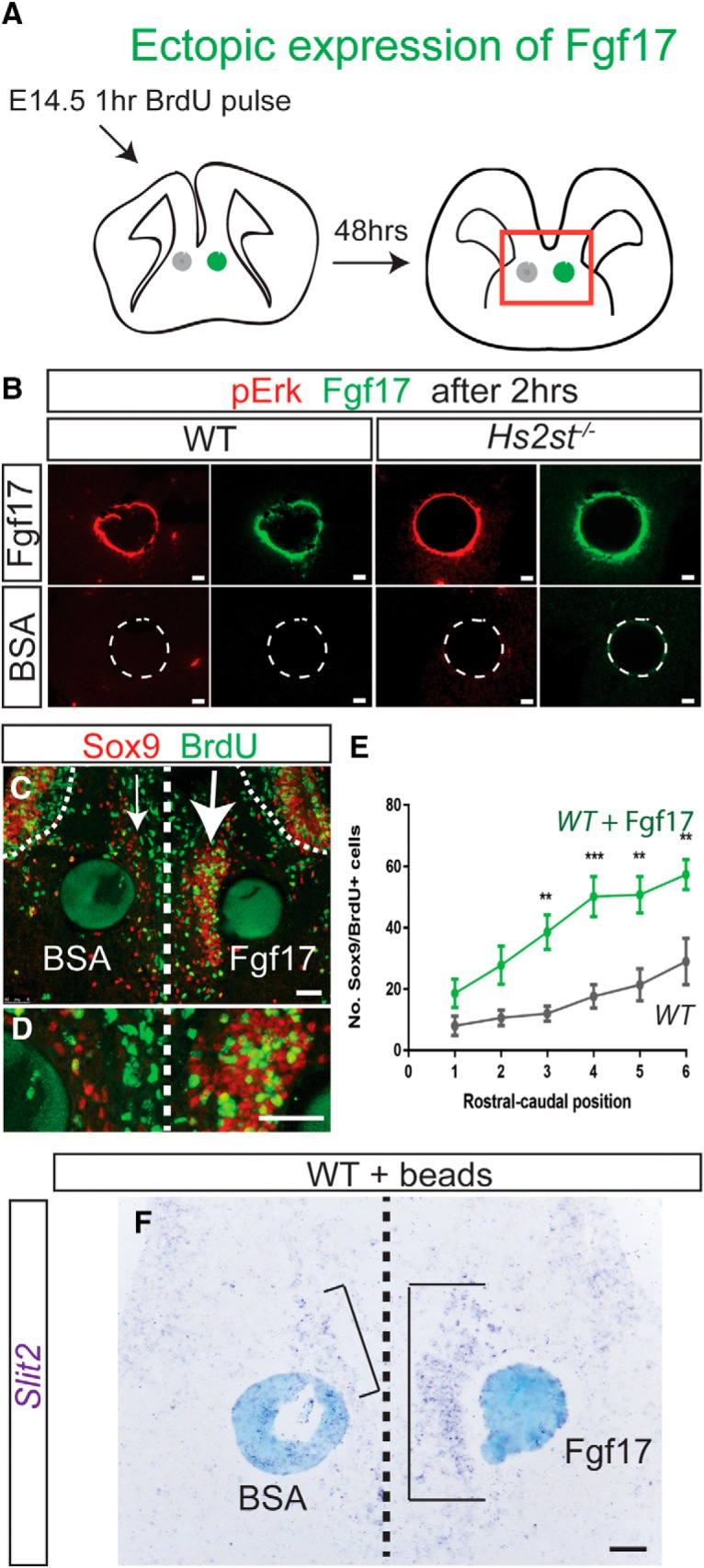
Fgf17-bead experiment. ***A***, Experimental outline of Fgf17 protein bead experiment in WT embryos. Pregnant females were injected at E14.5 with a BrdU pulse and CSB slices collected. One Fgf17 and one BSA bead were added to each side of the midline. ***B***, Fgf17 protein and pErk after 2 h in culture. In both WT and *Hs2st*^−/−^ CSB slices, Fgf17 and pErk are seen in tissue surrounding the Fgf17 bead. Staining for either is absent around the BSA bead (demarcated by dotted circle). ***C***, Immunofluorescence for BrdU and Sox9 was performed on WT slices after 48 h in culture; curved dotted lines indicate the basal edge of the VZ and straight dotted line indicates the midline. ***D***, Higher magnification of the arrowed regions in ***C***. ***E***, Quantification of Sox9^+^/BrdU^+^ double-labeled cells in WT CSB slice cultures with Fgf17 or BSA bead. The Fgf17 bead significantly increased the number of Sox9^+^/BrdU^+^ cells that exit the VZ and moved toward the IG (significant differences indicated on graph as **p < 0.05, ***p < 0.001) at the four caudal-most positions (*n* = 5 embryos; *F*_(1,48)_ = 65.63, *p* = 0.000000000155, two-way ANOVA, followed by *t* test with Sidak's correction for multiple comparisons for Fgf17-bead vs BSA-bead at each rostral-caudal position: position 1, *t*_(48)_ = 1.45, *p* = 0.63, *t* test; position 2, *t*_(48)_ = 2.36, *p* = 0.13, *t* test; position 3, *t*_(48)_ = 3.65, *p* = 0.0039, *t* test; position 4, *t*_(48)_ = 4.47, *p* = 0.0003, *t* test; position 5, *t*_(48)_ = 4.03, *p* = 0.0012, *t* test; and position 6, *t*_(48)_ = 3.89, *p* = 0.0018, *t* test). ***F***, *Slit2* expression in slices cultured with Fgf17 and BSA beads. Scale bars, 100 μm.

Our data suggest that the *Hs2st*^−/−^ phenotype stems from abnormally high levels of Fgf17 protein at the CSB, causing FGF/ERK hyperactivation and precocious somal translocation to the IG. This requires that *Hs2st*^−/−^ CSB cells are competent to respond to Fgf17 protein and that application of ectopic Fgf17 triggers precocious glial translocation, neither of which has been previously established. We redeployed the CSB *ex vivo* culture assay (Fig. 7) with the modification that beads soaked in either recombinant Fgf17 protein (Fgf17 treatment) or in BSA (control) were implanted into coronal slices of CSB region on either side of the midline (Fig. 9*A*). WT or *Hs2st*^−/−^ slices implanted with Fgf17 and BSA beads were cultured for 2 h before processing for Fgf17 (green signal) and pErk (red signal) double immunofluorescence (Fig. 9*B*). In both WT and *Hs2st*^−/−^ cultures, Fgf17 protein was detectable adjacent to the edge of the bead (green signal) and this activated ERK phosphorylation in a similar pattern (red signal) with no obvious differences between WT and *Hs2st*^−/−^, indicating that *Hs2st*^−/−^ CSB tissue is competent to respond to Fgf17 (Fig. 9*B*, top row). The lack of Fgf17 or pERK signal in the BSA control (Fig. 9*B*, bottom row) confirms Fgf17 antibody specificity and that pERK activation was specifically induced by exogenously applied Fgf17. We performed Sox9/BrdU analysis (exactly as described above for the FGFi experiments; Fig. 7) to assess the impact of experimentally introduced Fgf17 on astroglial translocation to the midline in WT CSB slices after 48 h in culture and the results were dramatic. The side with the Fgf17-bead showed many more Sox9^+^ (red) cells in the IG region (large arrow on right side of Fig. 9*C*) than the side with the BSA bead (smaller arrow on left side of Fig. 9*C*). Quantification of Sox9^+^;BrdU^+^ (yellow) cells (Fig. 9*D* shows higher magnification of IG region) confirmed a significant increase in astroglial translocation to the midline along the rostrocaudal axis on the side exposed to Fgf17 [Fig. 9*E*, cf. green (Fgf17) with black (control) lines]. An important function of IG glia is to secrete Slit2 and repulsively guide CC axons in the correct trajectory across the midline. At E16.5, *Slit2* mRNA is normally expressed in the IG region and, in *Hs2st*^−/−^ embryos, the midline *Slit2* expression domain is expanded (cf. Fig. 2*C*,*E* and *D*,*F*; *Slit2* expression domain is bracketed). Experimentally introduced Fgf17 is sufficient to phenocopy this aspect of the *Hs2st*^−/−^ phenotype in our *ex vivo* assay because the side exposed to the Fgf17 bead has a much larger *Slit2* domain than the BSA-treated side [cf. left (control) and right (Fgf17) bracketed areas in Fig. 9*F*], consistent with precocious translocation of excessive numbers of *Slit2*^+^ IG glia.

**Figure 10.**
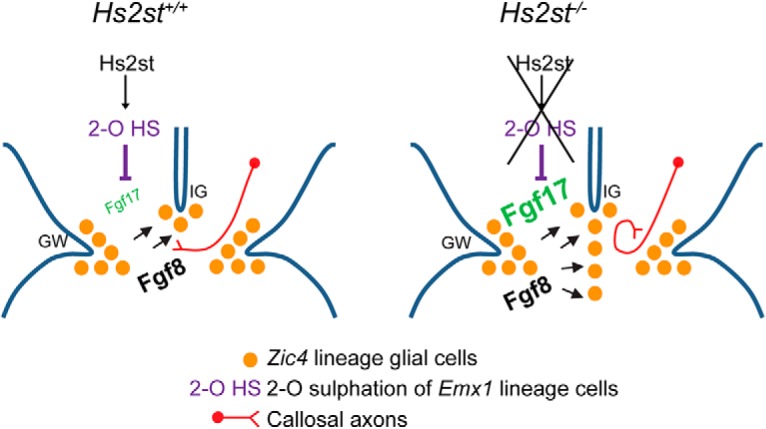
Model. Hs2st expressed in *Emx1*-lineage cells catalyzes 2-O HS sulfation (2-O HS) that in turn suppresses the levels of Fgf17 protein, but not Fgf8 protein, by an unknown mechanism at the CSB. *Zic4* lineage astroglial precursors respond to Fgf8 and Fgf17 protein by activating FGF/ERK signaling and translocating (black arrows) to the midline. This generates appropriate positioning of *Slit2*^+^ astroglia to guide CC axons across the midline. Loss of 2-O HS from the *Emx1* lineage selectively desuppresses Fgf17 protein levels leaving Fgf8 protein unaffected. This causes hyperactive FGF/ERK signaling and more *Zic4* lineage astroglial precursors translocate than normal with consequent blocking of CC axon midline crossing by the ectopic midline *Slit2*^+^ astroglia. *Zic4* lineage astroglial precursor cells do not need to express 2-O HS to respond to FGF signaling proteins and translocate to the midline.

We conclude that *Hs2st*^−/−^ CSB tissue is competent to respond to Fgf17 protein and abnormally high levels of Fgf17 protein are sufficient to phenocopy the *Hs2st*^−/−^ astroglial translocation phenotype consistent with the model presented in Figure 10.

### *Hs2st* selectively facilitates physical interaction between Fgf17 protein and HS

**Figure 11.**
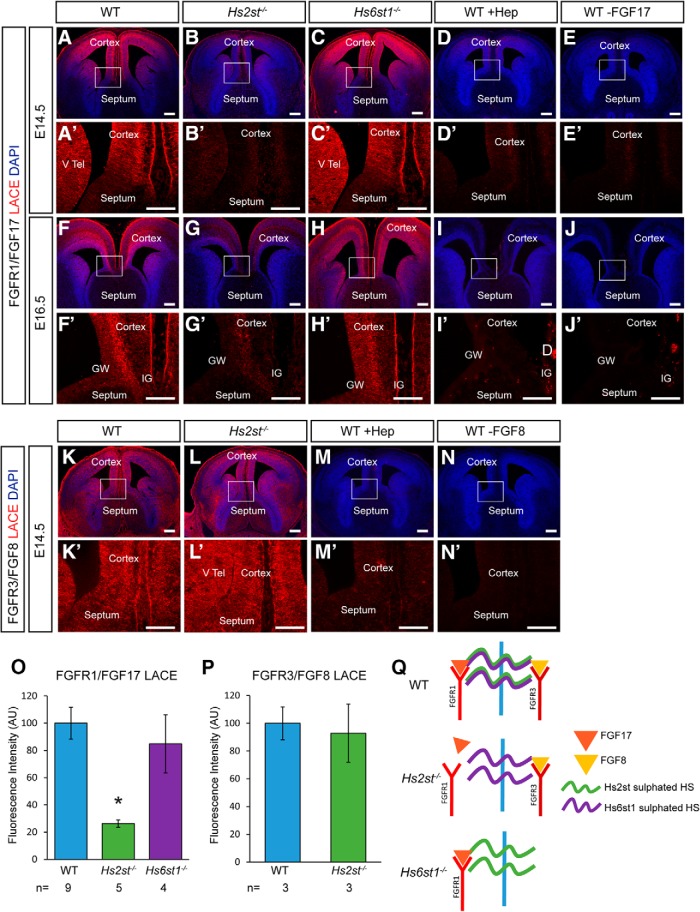
LACE assay for FGF:HS interactions. ***A***–***J***, ***O***, FGFR1/FGF17 LACE experiments on (***A***–***E***) E14.5 and (***F***–***J***) E16.5 telencephalic coronal sections through the CSB. ***A***, ***F***, WT. ***B***, ***G***, *Hs2st*^−/−^. ***C***, ***H***, *Hs6st1*^−/−^. ***D***, ***I***, WT sections pretreated with heparinitase to digest HS. ***E***, ***J***, WT sections with FGF17 omitted from the LACE assay. ***O***, Quantification of FGF17/FGFR1 LACE signal in WT (blue bar, *n* = 9 embryos), *Hs2st*^−/−^ (green bar, *n* = 5 embryos), and *Hs6st1*^−/−^ (purple bar, *n* = 4 embryos), showing a significant decrease (*p < 0.05 on graph) in *Hs2st*^−/−^ embryos *F*_(2,15)_ = 8.62, *p* = 0.0032, ANOVA, followed by *post hoc t* test: WT vs *Hs2st*^−/−^, *t*_(9)_ = 6.11, *p* = 0.014, *t* test; WT vs *Hs6st1*^−/−^, *t*_(5)_ = 0.63, *p* = 0.56, *t* test). ***K***–***N***, ***P***, FGFR3/FGF8 LACE experiments on E14.5 telencephalic coronal sections through the CSB. ***K***, WT. ***L***, *Hs2st*^−/−^. ***M***, WT sections pretreated with heparinitase to digest HS. ***N***, WT sections with FGF8 omitted from the LACE assay. ***P***, Quantification of FGF8/FGFR3 LACE signal in WT (blue bar, *n* = 3 embryos) and *Hs2st*^−/−^ (green bar, *n* = 3 embryos) showing no significant difference (*t*_(3)_ = 0.29, *p* = 0.76, *t* test). Numbers of embryos of each genotype analyzed indicated under bars. ***Q***, Summary diagram. FGFR1/FGF17/HS complex formation is equally supported by WT and *Hs6st1*^−/−^ HS but less so by *Hs2st*^−/−^ HS, whereas FGFR3/FGF8/HS complex formation is equally supported by WT and *Hs2st*^−/−^ HS, showing that the FGF17:HS physical molecular interaction is selectively dependent on 2-O HS sulfation. ***A′***–***N′***, Higher magnification showing the CSB region boxed in ***A***–***N*** (note that the DAPI channel is not shown in the higher-magnification images to improve visualization of the LACE signal). Scale bars, 200 μm.

Our *in vivo* data show that Hs2st suppresses the levels of Fgf17 protein and that this represents a selective interaction between Hs2st-mediated 2-O HS sulfation and Fgf17 protein levels *in vivo* because Hs2st does not suppress the levels of the closely related Fgf8 protein, and Hs6st1, which catalyzes 6-O HS sulfation, does not suppress Fgf17 protein levels (Clegg et al., 2014; Chan et al., 2017) (Fig. 8). However, these *in vivo* experiments do not resolve whether differential sulfation has a correspondingly direct selective effect on the physical interaction between HS and Fgf17. To test the hypothesis that Hs2st has a selective effect on the binding of Fgf17 protein to HS molecules, we turned to a biochemical assay, the LACE assay, which probes the physical interaction between HS and FGF proteins by quantifying the ability of endogenous HS in tissue sections to form Fgf:Fgfr:HS complexes with exogenously added Fgf protein and Fgfr ectodomain fused to an Fc tag for immunofluorescent detection (Allen et al., 2001; Chan et al., 2015). We used the Fgf17:Fgfr1 LACE assay to compare the binding of Fgf17 protein to HS in WT, *Hs2st*^−/−^, and *Hs6st1*^−/−^ CSB tissue at E14.5 and E16.5 to test the hypothesis that Fgf17:HS physical interaction is selectively sensitive to loss of 2-O HS sulfation in *Hs2st*^−/−^ tissue (Fig. 11*A–J*,*O*). We used the Fgf8:Fgfr3 LACE assay to compare the binding of Fgf8 protein to HS in WT and *Hs2st*^−/−^ CSB tissue at E14.5 to test the hypothesis that the Fgf8:HS physical interaction is insensitive to loss of 2-O HS sulfation in *Hs2st*^−/−^ tissue (Fig. 11*K–N*,*P*).

In both E14.5 and E16.5 WT tissue, the Fgf17:Fgfr1 and Fgf8:Fgfr3 LACE assays produced a strong LACE signal (Fig. 11*A*,*F*,*K*, with higher magnification of boxed areas enclosing CSB region shown in *A′*,*F′*,*K′*). Control experiments show this LACE signal was drastically reduced by pretreating the tissue with heparitinase to digest HS (Fig. 11*D*,*I*,*M*, with higher magnification of CSB in *D′*,*I′*,*M′*) or omitting Fgf17 or Fgf8 protein from the assay (Fig. 11*E*,*J*,*N*, with higher magnification of CSB in *E′*,*J′*,*N′*). Together, these controls confirm that the LACE signal provides a specific readout of the interaction between each FGF protein and HS molecules. To determine the effect of differential sulfation on the physical interaction between HS and Fgf8 or Fgf17, we investigated how the LACE signal was affected when the assay was performed on *Hs2st*^−/−^ and *Hs6st1*^−/−^ tissue. As predicted by our hypothesis, the binding of Fgf17 to HS is selectively sensitive to 2-O HS sulfation because we found that the Fgf17:Fgfr1 LACE signal was much weaker than WT in *Hs2st*^−/−^ tissue (cf. *B*,*B′*,*G*,*G′* and *A*,*A′*,*F*,*F′*), but similar to WT in *Hs6st1*^−/−^ tissue (cf. *C*,*C′*,*H*,*H′* and *A*,*A′*,*F*,*F′*). Quantification of Fgf17:Fgfr1 LACE signal intensity in Figure 11*O* shows a significant ∼4-fold reduction in *Hs2st*^−/−^ (green bar) compared with WT (blue bar), but no significant difference from WT in *Hs6st1*^−/−^ (purple bar). As predicted by our hypothesis that the binding of Fgf8 to HS is not sensitive to 2-O HS sulfation, we found that there was no difference in the Fgf8:Fgfr3 LACE signal between WT and *Hs2st*^−/−^ tissue (cf. Fig. 11*K*,*K′* and *L*,*L′*). Quantification of Fgf8:Fgfr3 LACE signal intensity in Figure 11*P* shows no significant difference between *Hs2st*^−/−^ (green bar) compared with WT (blue bar). These LACE results are summarized schematically in Figure 11*Q*, which shows that, of the five *HST* genotype and FGF ligand permutations tested, only the Fgf17:HS physical interaction was sensitive to the *Hs2st* genotype, as predicted by the hypothesis that 2-O HS sulfation has a specific effect on the ability of HS to bind Fgf17.

## Discussion

Embryonic CC development involves multiple cell and molecular events that ultimately guide callosal axons across the telencephalic midline to connect with their synaptic targets in the contralateral hemisphere. Three subpopulations of midline astroglia play pivotal roles in guiding callosal axons across the telencephalic midline. MZ glia facilitate fusion of the cerebral hemispheres and provide a substrate for crossing callosal axons, whereas *Slit2*^+^ IG and GW astroglia channel crossing axons into the correct path by Robo/Slit-mediated chemorepulsion (Shu and Richards, 2001; Bagri et al., 2002; Shu et al., 2003; Gobius et al., 2016). These astroglial populations originate from RGCs born in the VZ of the septal midline and either remain in the VZ at the GW or translocate in response to FGF signals, of which Fgf8 appears to be particularly important, to the pial surface of the telencephalic midline (MZ and IG astroglia) (Smith et al., 2006; Moldrich et al., 2010; Clegg et al., 2014; Gobius et al., 2016). Both Slit2^+^ IG and Slit2^−^ MZ astroglia are essential for CC development and both of these astroglial populations originate from the septal VZ *Zic4* lineage, so the lack of an overt CC phenotype in *Hs2st*^Fl/Fl^*;Zic4*^Cre^ embryos following conditional knock-out of *Hs2st* in the *Zic4* lineage indicates that neither MZ or IG astroglial precursors have a cell-autonomous requirement for *Hs2st* to translocate in appropriate numbers. In *Hs2st*^−/−^ embryos, there is an expansion of the *Slit2* expression domain at the CSB pial surface coinciding with increased Sox9^+^ glial cells and this is phenocopied by application of exogenous Fgf17 to *Hs2st*^+/+^ CSB *ex vivo*, strongly suggesting that increased numbers of Slit2^+^ glial cells at the midline reflect excessive GW→IG somal translocation enlarging the IG (current study). We cannot rule out the possibility that disrupted MZ glial translocation also contributes to the *Hs2st*^−/−^ phenotype, although this would not alter our conclusion that Hs2st plays a non-cell-autonomous role in the *Zic4*-lineage astroglial translocation phenotype. Our model (Fig. 10) posits that ectopic *Slit2*^+^ astroglia at the midline block the transit of CC axons. In principle, this could be tested by rescuing the CC axon midline crossing in *Hs2st*^−/−^*;Slit2*^−/−^ embryos (along similar lines to the *Slit2* genetic rescue of the *Hs6st1*^−/−^ phenotype that we reported in *Hs6st1*^−/−^*;Slit2*^−/−^ embryos; Conway et al., 2011). However, in contrast to the fully penetrant (100%) *Hs6st1*^−/−^ CC phenotype, the partial penetrance (∼50%) of the *Hs2st*^−/−^ CC phenotype introduces a confounding factor of distinguishing “rescued” from “unaffected” *Hs2st*^−/−^ embryos, a problem that would be compounded if only a proportion of embryos destined to be affected were rescued (Conway et al., 2011; Clegg et al., 2014), so a prohibitively large number of animals would be required to demonstrate a statistically significant rescue.

Eliminating HS (*Ext1* mutagenesis) compared with 2-O HS sulfation (*Hs2st* mutagenesis) from the same cell lineages allowed us to distinguish physiological functions generally attributable to HS from those specifically requiring 2-O HS sulfation by comparing the *Ext1* and *Hs2st* phenotypes. We found that, although *Zic4*-lineage cells were unable to support CC development when they lacked HS (*Zic4*^Cre^*;Ext1*^Fl/Fl^ embryos), there was no similar requirement for 2-O HS sulfation in the *Zic4* lineage (*Zic4*^Cre^*;Hs2st*^Fl/Fl^ embryos), indicating that *Zic4*-lineage cells require HS but that 2-O HS sulfation is dispensable for their contribution to CC development, specifically the ability of astroglial precursors to cell autonomously sense translocation signals. We found that HS and 2-O HS sulfation are both required in the *Emx1* lineage (*Emx1*^CreER^*;Ext1*^Fl/Fl^ and *Emx1*^CreER^*;Hs2st*^Fl/Fl^ embryos), although the axonal and astroglial phenotypes were not identical. Somewhat counter-intuitively, removing HS completely from the Emx1 lineage (*Emx1*^CreER^*;Ext1*^Fl/Fl^ embryos) has a less severe effect on the distribution of GFAP^+^ midline astroglia than preserving HS but specifically blocking it's 2-O sulphation (*Emx1*^CreER^*;Hs2st*^Fl/Fl^ embryos) with the more subtle disruption to HS causing the more pronounced accumulation of astroglia at the pial surface. We speculate that completely removing HS from the *Emx1* lineage results in a general destabilization of FGF protein gradients thus mitigating precocious somal translocation by *Zic4* lineage astroglial precursors (Qu et al., 2011, 2012; Shimokawa et al., 2011; Chan et al., 2017). The relatively normal midline astroglial organization in *Emx1*^CreER^*;Ext1*^Fl/Fl^ embryos poses the question of whether glial disorganization is a major contributor to their CC agenesis phenotype. In *Emx1*^CreER^*;Hs2st*^Fl/Fl^ embryos, the Probst bundles form right next to the midline, consistent with our hypothesis that ectopic Slit2^+^ astroglia at the midline are repelling CC axons from crossing the midline (Conway et al., 2011 and current study). In contrast, the Probst bundles in *Emx1*^CreER^*;Ext1*^Fl/Fl^ embryos form much more lateral to the midline at some distance from the IG, indicating that CC axons are misrouted at an earlier stage of their navigation than in *Emx1*^CreER^*;Hs2st*^Fl/Fl^ embryos. HS is required cell autonomously for navigating axons to respond to axon guidance molecules, including Netrin1 and Slit2 (Piper et al., 2006; Matsumoto et al., 2007). A plausible explanation is that, in *Emx1*^CreER^*;Ext1*^Fl/Fl^ embryos, the *Emx1*-lineage HS-deficient CC axons cannot respond appropriately to guidance cues that would normally guide them toward the midline and are already misrouted before they come under the influence of the midline astroglia. In contrast, *Hs2st*^−/−^ CC axons express HS lacking 2-O sulfation, which does not affect their ability to respond to guidance cues (current study), so they reach the midline but are prevented from crossing by the ectopic Slit2^+^ glia in the expanded IG.

Biochemical (LACE) data show that physical interaction between Fgf17 and HS is facilitated by Hs2st (but not Hs6st1) and that Hs2st facilitates physical interaction between HS and Fgf17 (but not Fgf8), suggesting a molecular mechanism underpinning Hs2st selectively suppressing levels of Fgf17 *in vivo* (Allen and Rapraeger, 2003; Clegg et al., 2014; Chan et al., 2015, 2017; current study). We speculate that Hs2st exerts its selective effect on Fgf17 protein levels because HS lacking 2-O HS sulfation has reduced affinity for Fgf17 (but not Fgf8), so increasing the half-life of Fgf17 (but not Fgf8) in the ECM by selectively reducing the rate that Fgf17 protein is cleared by HS-mediated receptor-mediated endocytostis of canonical FGFs while leaving Fgf8 unaffected (Yu et al., 2009). Our conditional mutagenesis experiments clearly demonstrate there is non-cell-autonomous requirement for *Hs2st* in astroglial precursor translocation in *Emx1*^CreER^*;Hs2st*^Fl/Fl^ embryos; however, the reduced efficiency of HS:Fgf17:Fgfr1 complex formation in the *Hs2st*^−/−^ LACE assay implies that *Hs2st* might also play a cell-autonomous role in the response to Fgf17 protein. We speculate that, even if *Hs2st*^−/−^ astroglial precursors are less sensitive to Fgf17 than their WT counterparts, their translocation to the midline is primarily driven by Fgf8, so it is not significantly affected in *Zic4*^Cre^*;Hs2st*^Fl/Fl^ embryos. A putative reduced sensitivity of *Hs2st*^−/−^ astroglial precursor cells to Fgf17 also begs the question of how elevated Fgf17 could trigger precocious glial translocation in *Hs2st*^−/−^ embryos. The Fgf17 bead assay experiment shows that *Hs2st*^−/−^ cells retain competence to respond to Fgf17 by phosphorylating ERK and LACE data show that HS devoid of 2-O HS sulfation still interacts with Fgf17, albeit with reduced efficiency. Therefore, the explanation that best fits our experimental data is that increased Fgf17 protein levels in *Hs2st*^−/−^ embryos override any reduction in competency of *Hs2st*^−/−^ cells to respond to Fgf17 protein and the net effect is elevated FGF/ERK signaling and consequent precocious astroglial translocation.

This study makes two major novel contributions to our understanding of the cell and molecular roles of differential HS sulfation in the regulation of forebrain development. First, a primary cellular role of 2-O HS sulfation *in vivo* is not to modulate the competence of astroglial precursor cells to respond to translocation signals by a cell-autonomous mechanism (as would be predicted by the classic role for HS in modulating the formation of the FGF:FGFR:HS receptor complex on the surface of responding cells), but instead to regulate the supply of translocation signals to astroglial precursors by a non-cell-autonomous mechanism. Second, the interaction between 2-O-sulfated HS and Fgf17 protein is selective because it does not apply to the closely related Fgf8 protein or to 6-O HS sulfation catalyzed by Hs6st1. The most parsimonious explanation linking these cell and molecular events is that higher than normal levels of Fgf17 protein at the CSB of *Hs2st*^−/−^ embryos causes the precocious astroglial precursor translocation phenotype and subsequent misrouting of CC axons (Fig. 10). Our rescue of the *Hs2st*^−/−^ precocious astroglial precursor translocation phenotype *ex vivo* by generic pharmacological inhibition of FGF signaling with SU5402 directly supports the hypothesis that hyperactive FGF/ERK signaling causes the phenotype. Given the well known role of FGF/ERK signaling in triggering astroglial precursor translocation to the IG, our findings that exogenously applied Fgf17 protein is sufficient to phenocopy the *Hs2st*^−/−^ astroglial precursor translocation phenotype and that *Hs2st*^−/−^ CSB cells activate ERK in response to Fgf17 protein, it is extremely unlikely that increased Fgf17 protein levels *in vivo* would not result in ERK hyperactivation and consequent precocious astroglial precursor translocation in *Hs2st*^−/−^ embryos. However, the current study does not provide formal proof that the elevated levels of Fgf17 protein are solely responsible for the FGF/ERK hyperactivation or precocious astroglial precursor translocation phenotypes in *Hs2st*^−/−^ embryos and we were unable to design an experiment that could further discriminate between the functions of Fgf17 and Fgf8 and directly test functional selectivity of Hs2st for Fgf17 in this context. We considered using a classic rescue experiment strategy by genetically reducing *Fgf17* dosage in *Hs2st*^−/−^ embryos (*Fgf17*^−/−^*;Hs2st*^−/−^ rescue), but elected not to because, at best, it would provide equivocal evidence either for or against the hypothesis that 2-O-sulfated HS interacts selectively with Fgf17 protein. FGF/ERK hyperactivation caused by overexpression of a particular FGF protein can be rescued by any experimental manipulation that restores ERK signaling to normal levels and not uniquely by restoring the levels of the FGF protein that underpins the phenotype. Specifically, reducing *Fgf17* dosage could elicit a rescue of ERK hyperactivation and collateral phenotypes at the *Hs2st*^−/−^ CSB by reducing FGF/ERK signaling output regardless of whether abnormally high Fgf17 bioavailability was the primary cause. Analogously, we interpret rescue of the *Hs6st1*^−/−^ precocious astroglial precursor translocation phenotype in *Hs6st1*^−/−^*;Fgf8*^neo/neo^ embryos as evidence that *Hs6st1* normally acts to keep FGF/ERK signaling in check rather than as evidence for a selective genetic interaction between *Fgf8* and *Hs6st1* (Clegg et al., 2014). Conversely, failure to rescue the *Hs2st*^−/−^ phenotype in *Fgf17*^−/−^*;Hs2st*^−/−^ embryos (or using other methods to reduce Fgf17 protein levels or functionality) would not falsify the hypothesis that increased Fgf17 bioavailability caused the *Hs2st*^−/−^ phenotype because there are several alternative explanations. When we used a similar strategy in a similar context to rescue the *Hs6st1*^−/−^ astroglial precursor precocious translocation phenotype by genetically reducing *Fgf8* dosage, the rescue was only successful in a minority of isogenic *Hs6st1*^−/−^*;Fgf8*^neo/neo^ embryos and a likely explanation is that compensatory mechanisms act when *Fgf* gene dosage is manipulated (Clegg et al., 2014). Such compensation will generate false-negative results, making it unsafe to interpret unrescued *Fgf17*^−/−^*;Hs2st*^−/−^ embryos as falsifying the hypothesis that the phenotype is underpinned by excess Fgf17 protein. There are additional technical confounds that could lead to false negatives because a rescue likely requires precise restoration of normal Fgf17 protein levels (so no rescue could reflect technical failure to restore Fgf17 protein levels to normal) and, in any case, the CC phenotype of *Fgf17*^−/−^ embryos has not been thoroughly characterized, so *Hs2st*^−/−^*;Fgf17*^−/−^ phenotypes may be problematic to interpret (Cholfin and Rubenstein, 2007, 2008). In addition to not being decisive for or against selectivity, demonstrating genetic interaction between *Hs2st* and *Fgf17* would not provide insight into whether the interaction was molecularly direct, in contrast to the biochemical LACE data that we present in the current study.

The closely related *Fgf8* subfamily members *Fgf17* and *Fgf8* are both transcribed by cells in the CSB region, yet have different roles in forebrain development with available evidence, although this does not rule out a role for *Fgf17*, suggesting that *Fgf8* is the primary driver of astroglial precursor translocation required for CC development (Cholfin and Rubenstein, 2007, 2008; Moldrich et al., 2010; Toyoda et al., 2010; Gobius et al., 2016). The independent suppression of Fgf17 and Fgf8 protein levels by HS modified by Hs2st and Hs6st1, respectively, may have facilitated the evolution of this system by providing a mechanism to tilt the Fgf17:Fgf8 protein balance to give Fgf8 the more dominant role in regulating astroglial precursor translocation (Clegg et al., 2014; Chan et al., 2017, current study). In this sense, there are parallels to other negative regulatory strategies, such as microRNAs that function by protecting cells from the expression of particular proteins that would be detrimental if expressed.
